# Screening and Identification of Differential Ovarian Proteins before and after Induced Ovulation via Seminal Plasma in Bactrian Camels

**DOI:** 10.3390/ani11123512

**Published:** 2021-12-09

**Authors:** Qi Wang, Quanwei Zhang, Yina Li, Xingxu Zhao, Yong Zhang

**Affiliations:** 1 College of Veterinary Medicine, Gansu Agriculture University, Lanzhou 730070, China; wangqi@gsau.edu.cn (Q.W.); lyn9097@163.com (Y.L.); 2 College of Life Science and Technology, Gansu Agriculture University, Lanzhou 730070, China; zhangqw@gsau.edu.cn

**Keywords:** iTRAQ, induced ovulation, ovary, Bactrian camel

## Abstract

**Simple Summary:**

Camelidae are induced ovulators whose ovulation is tightly regulated by multiple factors. Understanding the biological mechanisms underlying follicular development, hormone secretion, and ovulation requires investigating the potential molecular pathways involved in these mechanisms. However, little is known about these molecular pathways in Bactrian camels. To screen and identify candidate biomarkers after seminal plasma (SP)-induced ovulation in the ovaries, we performed comprehensive proteomic and molecular biological analyses of the ovaries from camels that were intramuscularly injected with either seminal plasma or phosphate-buffered saline. Identification of these candidate biomarkers will enable a better understanding of reproduction in Bactrian camels. Our findings suggest candidate proteins for further studies on the molecular mechanisms of induced ovulation.

**Abstract:**

Camelidae are induced ovulators whose ovulation is tightly regulated by multiple factors. Understanding the biological mechanisms underlying follicular development, hormone secretion, and ovulation requires investigating the potential molecular pathways involved. However, little is known about these pathways in Bactrian camels. To screen and identify candidate biomarkers after inducing ovulation, this study performed comprehensive proteomic and molecular biological analyses of the ovaries from two camel groups (*n* = 6). We identified 5075 expressed ovarian proteins, of which 404 were differentially expressed (264 upregulated, 140 downregulated) (*p* < 0.05 or *p* < 0.01), in samples from plasma-induced versus control camels. Gene ontology annotation identified the potential functions of the differentially expressed proteins (DEPs). These results validated the differential expression for a subset of these proteins using Western blot (*p* < 0.05) and immunofluorescence staining. Three DEPs (FST, NR5A1, and PRL) were involved in neurochemical signal transduction, as well as endocrine and reproductive hormone regulatory processes. The Kyoto Encyclopedia of Genes and Genomes analysis indicated the involvement of several pathways, including the calcium, cAMP, gonadotropin-releasing hormone, MAPK, and neuroactive ligand–receptor signaling pathways, suggesting that induced ovulation depends on the hypothalamic–pituitary–ovarian axis. Identifying these candidate biomarkers enables a better understanding of Bactrian camel reproduction. Ovarian proteomic profiling and the measurement of selected proteins using more targeted methods is a promising approach for studying induced-ovulation mechanisms.

## 1. Introduction

The Bactrian and dromedary camel, alpaca, llama, Guanaco and vicuna, belong to the family Camelidae [1]. Camels live in extreme desert environments in Africa and Asia, and their adaptations to arid conditions include hydropenia and a tolerance of temperatures exceeding 40 °C [2,3]. Gonadotropin-releasing hormone (GnRH) and luteinizing hormone (LH) are critical regulatory factors of the neuro-endocrine system, which controls ovulation [4]. Two categories of ovulation, spontaneous and induced, have been classified in species, based upon the release of GnRH/LH. In spontaneous ovulators, such as cattle, goats, horses, monkeys, pigs, sheep, and most rodents, perennial or seasonal estrus leads to regular ovulation. In induced ovulates, such as alpaca, Bactrian camels and dromedary camels, cats, ferrets, llamas, and rabbits, neural signals elicited by the act of copulation trigger hypothalamic GnRH secretion, which induces preovulatory LH release from the pituitary gland [5]. Although different stimuli (olfactory, tactile, and visual) have been associated with eliciting/facilitating ovulation in induced ovulators [6,7], the physical stimulation of penile intromission or cervical stimulation plays a pivotal role in triggering the LH surge and subsequent ovulation [8]. Studies have shown that llama seminal plasma (SP) contains active ovulation-inducing factors (OIFs) similar to GnRH [9]. Zhao et al. isolated and purified OIFs from semen and found that a single intramuscular or intravaginal administration of male seminal plasma (SP) induced an LH surge, followed by ovulation in Bactrian camels [10]. Another study compared the effects of intravaginally administering fresh and frozen semen, SP, washed spermatozoa, and intramuscularly injecting SP on ovulation rates in Bactrian camels. This work showed that ovulation occurred in most females that were inseminated with whole semen, regardless of whether the semen contained frozen spermatozoa [11].

Studies have isolated and purified an OIF from other camelids (alpacas and llamas) [12], and determined the protein structure of this OIF by X-ray crystallography [13]. Other studies found that camel OIFs directly or indirectly regulate ovulation via the hypothalamic–pituitary–gonadal axis [14]. However, induced ovulation in Bactrian camels is highly complex, involving coordinated regulation by the endocrine, reproductive, and nervous systems, and by coding and regulating genetic information. The mechanism of induced ovulation in Bactrian camels remains unclear. Therefore, in this study, we used the isobaric tags for relative and absolute quantification (iTRAQ) technology to screen ovulation-inducing proteins via bioinformatics, and verified key target proteins via PCR, immunoblotting, and immunofluorescence staining to identify potential molecular regulatory mechanisms. This work provides a foundation for future research on induced ovulation and reproductive potential in Bactrian camels.

## 2. Materials and Methods

### 2.1. Animals and Handling Conditions

The study was conducted at the breeding base farm in Zhangye City. Nine Bactrian camels (three males and six females) were kept in a pasture and provided supplementary hay, feed pellets, and Italic water. All tissue samples were collected in strict accordance with the ethical guidelines approved by the Animal Care Commission of the College of Veterinary Medicine, Gansu Agriculture University (ethical approval number: GSAU-AEW-2018-0128).

### 2.2. Semen Collection and SP Preparation

Semen was collected from three mature (5–7 year-old) male Bactrian camels two to three times weekly for 3 months, using an artificial vagina inserted into a latex phantom as previously described [15]. Sixty ejaculates were obtained, with an average volume of 4 mL per ejaculate. Briefly, ejaculates were centrifuged at 1500× *g* for 30 min to remove spermatozoa. Sperm-free SP was pooled in 5–6-mL aliquots, a drop of supernatant was evaluated microscopically to confirm the absence of cells. If spermatozoa were detected, the sample was centrifuged and reevaluated until no spermatozoa were detected, and then stored at −20 °C. Sperm-free samples were stored at −80 °C for long-term storage.

### 2.3. SP Injection and Sample Collection

The 5–7 year-old female camels were examined daily by manual touch and transrectal ultrasonography using an ultrasound scanner (MyLab 30, Esaote; Maastricht, The Netherlands). Bactrian camels with growing preovulatory follicles (6–12 mm) were assigned to either the control group (intramuscularly injected with 5 mL of phosphate-buffered saline [PBS]; *n* = 3) or the treatment group (intramuscularly injected with 5 mL SP; *n* = 3). After injection, blood was collected every 4 h, and follicular development was detected via transrectal ultrasonography. After 48 h, the camels were slaughtered, and samples were collected.

### 2.4. Samples Preparation and Collection

Hypothalamus, pituitary, pineal gland, ovarian, uterine, and oviduct samples were obtained immediately after slaughtering (three treated and three control groups). Some samples were fixed for histomorphological observation; others were immediately stored in liquid nitrogen at −80 °C.

### 2.5. iTRAQ Labeling and SCX Fractionation and LC-Electrospray Ionization MS/MS

Two hundred milligrams of peptide mixture from each ovarian sample was labeled with iTRAQ reagent (Applied Biosystems), per the manufacturer’s instructions. iTRAQ-labeled peptides were fractionated via SCX chromatography using the AKTA Purifier system (GE Healthcare). LC-MS/MS analysis was performed on a Q Exactive mass spectrometer (Thermo Scientific), coupled to an Easy nLC (Proxeon Biosystems, now Thermo Fisher Scientific) for 60, 120, or 240 min (times were determined in a pilot study). The mass spectrometer was operated in the positive-ion mode. MS data were acquired using a data-dependent top 10 method, with the most abundant precursor ions from the survey scan (300–1800 *m*/*z*) being dynamically chosen for HCD fragmentation. The experimental process and data analysis were performed as previously described [16].

MS/MS spectra were searched using the MASCOT engine (Matrix Science, London, UK; version 2.2), embedded into Proteome Discoverer 1.4 (Thermo Fisher Scientific Inc., San Jose, CA, USA). The following parameters were set. Peptides were matched against the Bactrian camel database in NCBI with a peptide mass tolerance: 20 ppm, MS/MS tolerance: 0.1 Da, enzyme: trypsin, missed cleavage: 2, fixed modifications: carbamidomethyl (C), iTRAQ 8-plex (K), and iTRAQ 8-plex (N-term), variable modification: oxidation (M), search: thorough, and false discovery rate: b 0.01. Protein ratios were calculated as the median of only unique peptides of the protein quantification. All peptide ratios were normalized to the median protein ratio, with a median protein ratio of 1 after normalization. The ProGroup algorithm was used to eliminate redundancy. The *p*-values were calculated using a t-test. Statistical analysis was performed using SPSS version 22.0 (SPSS Inc., Chicago, IL, USA).

### 2.6. Hormone Analysis

Peripheral blood was collected at 0, 4, 8, 12, 24, 36, and 48 h, and the serum was then isolated to measure hormone levels. The estradiol (E2), follicle-stimulating hormone (FSH), gonadotropin-releasing hormone (GnRH), luteinizing hormone (LH), progesterone (PROG), and prolactin (PRL) contents were assayed and measured using radioimmunoassay (RIA) methods, as per Greenwood et al. [17]. The serum samples were incubated at 37 °C for 1–1.5 h, then hormone radioactive markers were added and incubated for another 1–1.5 h, then for another 10–12 h. After incubation, the precipitate was harvested and counted in a Packard Gamma Spectrometer for the measurement of bound radioactivity. All samples from both treatment groups were always assayed in the same RIA to avoid interassay variation, and to enable comparisons. Each experiment was repeated in its entirety at least three times.

### 2.7. Western Blot

Total protein was isolated from camel tissues using lysis buffer (Solarbio, Beijing, China) and quantified using a BCA protein assay kit (Boster). Samples equivalent to 50 μg of protein were resolved using sodium dodecyl sulfate-polyacrylamide gel electrophoresis (SDS-PAGE), and were electrotransferred to PVDF membranes (Millipore CAT, Billerica, MA, USA). The membranes were blocked for 2 h in Tris–HCl buffer containing 5% (*w*/*v*) non-fat milk powder, then were incubated overnight at 4 °C with the following primary antibodies from mice or rabbits: ACTB (1:5000, Proteintech, China), CAMK2G (1:1000, Proteintech), FST (1:1000, Proteintech), NR5A1 (1:500, Proteintech), PAK1 (1:1000, Santa Cruz, USA), PRL (1:1000, Santa Cruz), STAG2 (1:1000, Santa Cruz), TGFB2 (1:500, Santa Cruz), Wnt2b (1:500, Santa Cruz), GAPDH (1:5000, Proteintech). Membranes were then washed three times with PBS containing 0.1% Tween 20, incubated with goat anti-mouse/anti-rabbit IgG conjugated to horseradish peroxidase for 2 h at room temperature (1:5000, Bioss, Beijing, China), then washed three times with PBS containing 0.1% Tween 20. All immunoblot assays were performed at least three times. Optical densities of the bands were quantified, and membranes were scanned using Image-Pro plus 6.0 (Media Cybernetics Co., Rockville, MD, USA).

### 2.8. Quantitative Real-Time PCR (qRT-PCR) Analysis

Total RNA was extracted from tissues using the TransZol Up reagent (Invitrogen, Carlsbad, CA, USA), per the manufacturer’s instructions. After treatment with DNase, RNA was reverse transcribed using a RevertAid First-Strand cDNA Synthesis Kit (TransGen, Beijing, China), per the supplier’s instructions. Expression of the housekeeping gene GAPDH was used as an intratissue control. Table 1 lists the primers used. qPCR was conducted as reported previously [18]. 

### 2.9. Immunofluorescence Staining

Hematoxylin–eosin (HE) and immunohistochemical staining were performed as previously described [19]. For immunofluorescence detection, the sections were routinely dewaxed and hydrated, and antigens were retrieved by microwave heating. Sections were treated with an autofluorescence quencher (Servicebio, Wuhan, China) and incubated with 5% (*w*/*v*) bovine serum albumin for 30 min at room temperature. Next, sections were incubated with primary antibody (1:100; Santa Cruz or 1:150, Proteintech) overnight at 4 °C. After being washed with PBS, the sections were incubated with FITC- or ALEX-conjugated goat anti-mouse IgG (1:200; Abcam, Waltham, MA, USA) at room temperature for 1–1.5 h in the dark. The nuclei were stained with 4,6-diamidino-2-phenylindole (DAPI). Finally, the antifluorescence quencher was used to seal the sections. Images were observed and obtained using a fluorescence microscope (LSM800, ZEISS, Oberkochen, Germany).

### 2.10. Data Analysis

PCR and western blotting data are expressed as the mean ± standard deviation using Origin 8.6 (OriginLab, CA, USA). Data were analyzed via the Student’s *t*-test (between two groups) or a one-way analysis of variance (within multiple groups). *p <* 0.05 was considered statistically significant.

## 3. Results

### 3.1. Follicle Size and Serum Hormone Levels

**Figure 1:**
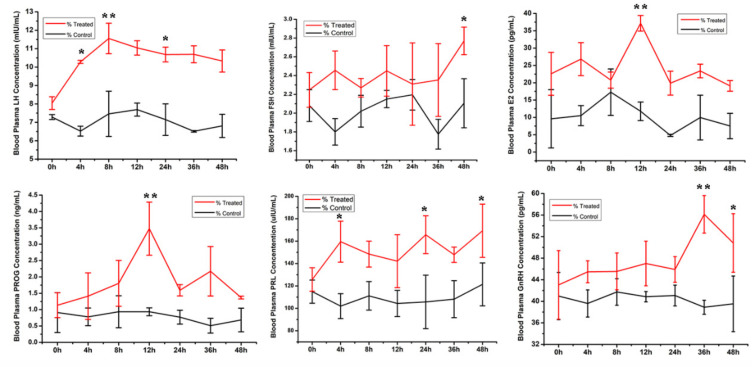
Serum hormone levels (LH, FSH, estradiol (E2), follicle-stimulating hormone (FSH), gonadotropin-releasing hormone (GnRH), progesterone (PROG) and prolactin (PRL) in the treated and control groups were detected by radioimmunity assay. Data are presented as mean ± SD (*n* = 3, * *p* < 0.05, ** *p* < 0.01).

Follicles of more than 10.0 mm diameter were collected; the average follicular diameter was 10.0 ± 1.5 mm. After 30 h, follicles in the treated group had decreased in volume, and follicular diameter decreased to approximately 7.0 ± 1.0 mm at 48 h. In the control group, the follicular diameters that were measured via B-ultrasound were unchanged (Figure S1). RIA results revealed that the circulating LH level was higher in the treated group than in the control group, with the highest LH level at 4 h, followed by a slow decrease in the treated group, compared with almost no change observed in the control group. The FSH level was also higher in the treatment group than in the control group. Additionally, the FSH level slowly increased with time in the treated group, whereas in the control group, it tended to decrease at 4 h and 36 h, and remained constant at other times. Serum E_2_ had increased in the treated group when compared with that of the control group, with the highest levels observed at 12 h, which were significantly higher than that at other time points. Serum PROG was higher in the treatment group than in the control group, and the PROG level peaked at 12 h, which was significantly higher than at other times. GnRH increased slowly from 0–24 h, with the highest concentration observed at 36 h. However, GnRH levels were relatively constant in the control group and did not change significantly (Figure 1).

### 3.2. Protein Profiling and Identification of Differentially Expressed Proteins

**Figure 2:**
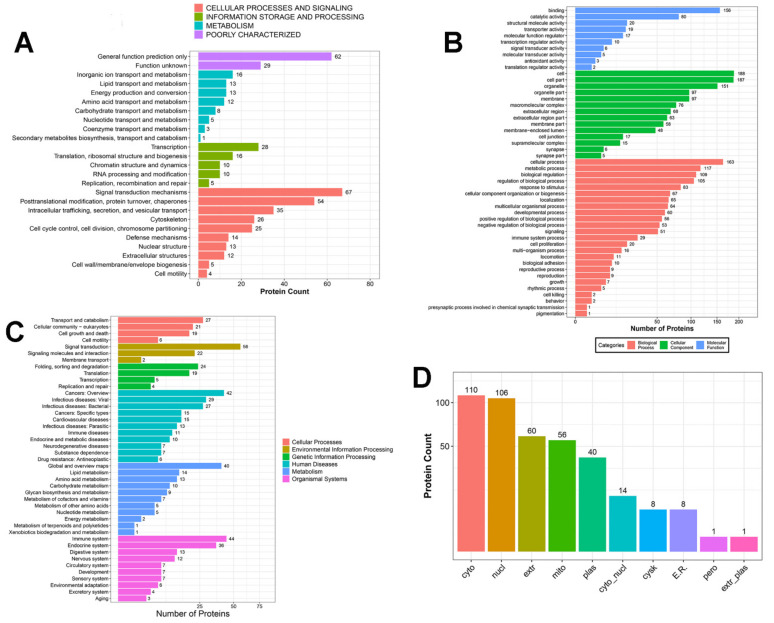
Annotation of differentially expressed proteins (DEPs). (**A**) Bar plot of KOG analysis of the DEPs. (**B**) Gene ontology (GO) annotation and functional classification of ovarian DEPs. GO terms are given for the subcellular location, biological process, cellular component, and molecular function. (**C**) KEGG Pathway analysis for the significantly enriched ovarian DEPs. (**D**) Bar plot of the predicted subcellular localization of DEPs; *x*-axis displays subcellular structures; *y*-axis displays protein count.

Ovarian samples from Bactrian camels in the treated and control groups were compared using an iTRAQ experiment. This study used the log2 of the protein’s fold change in expression and the log10 of its *p*-value. After processing the MS/MS spectra using Mascot software, 23,786 unique peptides were mapped to 5075 proteins. Of these, 404 DEPs were assigned to 25 clusters of orthologous groups (COG) categories, which included “signal transduction mechanisms” (67, 16.58%), “general function prediction only” (62, 15.35%), and “posttranslational modification, protein turnover, chaperones” (54, 13.37%; Figure 2A).

Using 0.8-fold and 1.2-fold changes as the cut-off values (*p* < 0.05), 404 proteins were differentially expressed between the treated and control groups; 264 were upregulated, and 140 were downregulated (Table 2). GO analysis showed that certain proteins were involved in cellular processes (40.35%), metabolic processes (28.96%), biological regulation (26.98%), and the reproduction and reproductive processes (2.23%). Proteins were also predicted to be components of cell structures (46.53%), organelles (37.37%), membranes (24.00%), and synapses (1.69%). Some were binding proteins (38.61%), while others were assigned as structural molecules (4.95%), those with catalytic activity (19.80%), and signal transducers (1.49%) (Figure 2B). The Kyoto Encyclopedia of Genes and Genomes (KEGG) functional analysis indicated that the ovarian DEPs were involved in cellular processes, environmental information processing and metabolism, and organismal systems, including 277 pathways. Neurological and reproductive endocrine pathways, such as the cAMP, MAPK, neuroactive ligand–receptor, phosphoinositide-3-kinase/Akt, NOD-like receptor, and transforming growth factor-beta signaling pathways, were upregulated or downregulated to various extents (Figure 2C). Subcellular localization analysis indicated that the DEPs are found in the cytoplasmic (110, 27.23%), nuclear (106, 26.24%), extracellular (60, 14.85%), mitochondrial (56, 13.86%), plasma (40, 9.90%), cytoplasmic and nuclear (14, 3.47%), cytoskeletal (8, 1.98%), endoplasmic reticular (8, 1.98%), peroxisomal (1, 0.25%), and extracellular and plasma (1, 0.25%) compartments (Figure 2D).

### 3.3. Protein–Protein Interaction (PPI) Network Analysis

**Figure 3:**
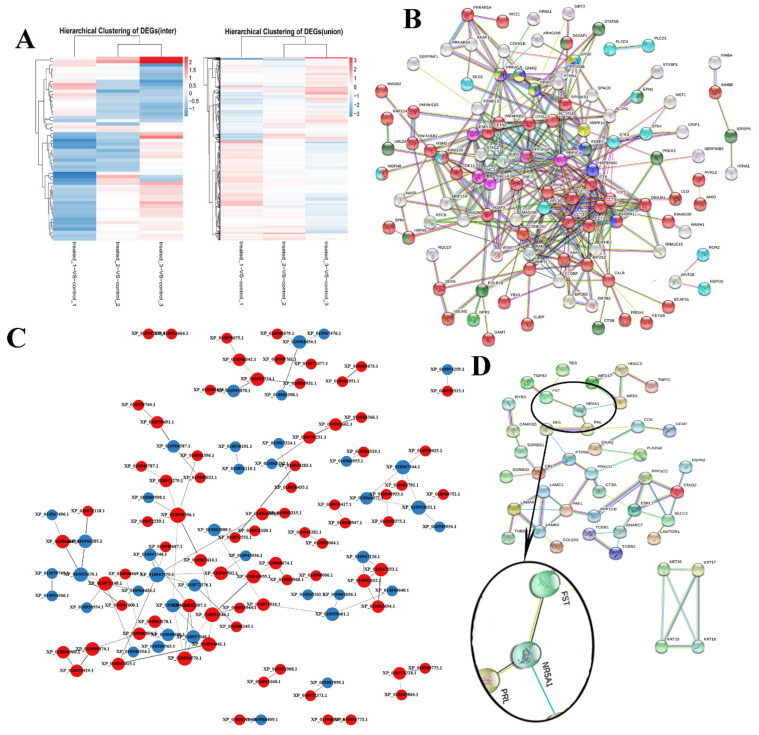
Clustering and protein–protein interaction (PPI) network analysis. (**A**) Hierarchical clustering of differentially expressed proteins (DEPs). Clustering was based on ovarian protein expression levels in the Bactrian camel. (**B**) Analysis of PPI network proteins related to the reproduction and reproductive processes. (**C**) Analysis of the PPI network for the ovarian DEPs. (**D**) Analysis of the PPI network for hormone and ovulation regulation related proteins in the ovarian DEPs.

The study grouped the 404 DEPs by their fold change in expression and their expression levels across each sample on a heatmap, with red representing significantly upregulated proteins, blue representing significantly downregulated proteins, and off-white representing proteins with no expression changes (Figure 3A). In total, 133 of the 5075 identified proteins were assigned to the reproduction and reproductive processes cluster and were used to generate a PPI network via the String online database and Cytoscape software (Figure 3B). We generated a PPI network of the 404 DEPs (264 upregulated and 140 downregulated in the treatment group) and identified 100 nodes/DEPs with 103 edges (Figure 3C). Thirty three proteins were related to the endocrine system, nineteen to reproduction and reproductive processes, and seventeen to neural signal transduction (Figure 3D). Further PPI analysis revealed 44 nodes and 77 edges in the PPI network, including the FST, NR5A1, PRL, CAMK2G, PAK1, PRKCD, PPP1CB, PPP1CC, and STAG2 central proteins. FST and NR5A1 are directly involved in steroid hormone synthesis and secretion. PRL-NR5A1-FST was mainly in the PPI network and contained four nodes and six edges. The KRT39-KRT19-KRT17-KRT18 cluster was associated with the estradiol receptor signaling pathway.

### 3.4. Expression Profile Verification

**Figure 4:**
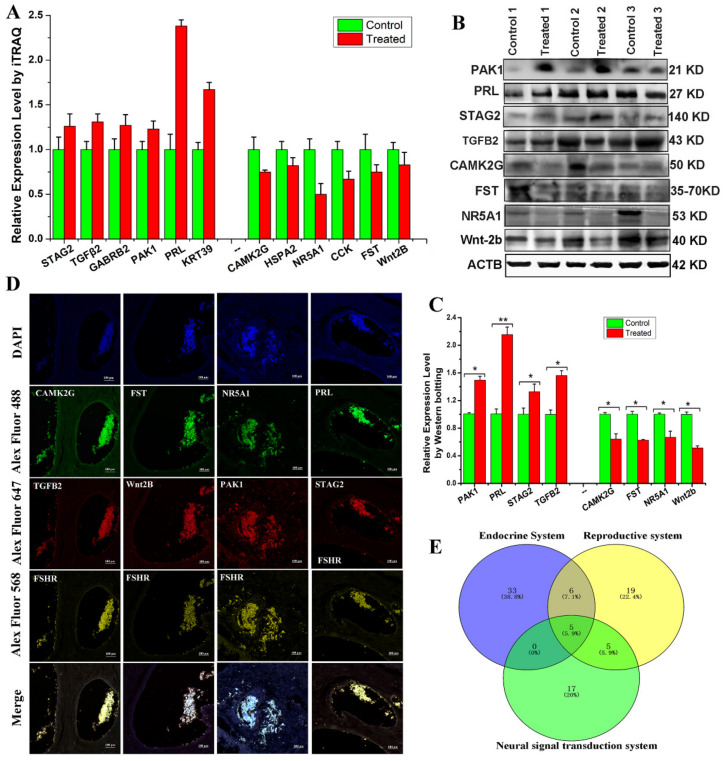
Validation of differentially expressed proteins (DEPs). (**A**) Protein expression levels detected in ovarian protein extracts from Bactrian camels via iTRAQ LC-MS/MS. (**B**) Western blot analysis of PAK1, PRL, STAG2, TGFB2, CAMK2G, FST, NR5A1, and Wnt2b in ovarian protein extracts from Bactrian camels. (**C**) Integrated optical density analysis of the Western blots using ImageJ. Data are presented as the mean ± standard deviation; * *p* < 0.05, ** *p* < 0.01. (**D**) Venn diagram of the DEPs using multiple comparisons of the endocrine system, reproductive system and neural signal transduction system groups. (**E**) Immunofluorescence staining to localize the expression patterns of the DEPs in the ovarian samples.

To verify the reliability of our iTRAQ results (Figure 4A), this study selected eight DEPs that were associated with reproduction, steroid hormone synthesis, and secretion for Western blot analysis. The results showed that GABRB2, KRT39, PAK1, PRL, STAG2, and TGF-β2 were abundantly expressed and upregulated, while CAMK2G, CCK, FST, HSPA2, NR5A1, and Wnt2b were downregulated in ovarian tissue from treated Bactrian camels. Western blotting data revealed that all eight DEPs showed expression patterns similar to those of the iTRAQ data, demonstrating the reliability of the iTRAQ data (Figure 4B,C). We used multiplex immunofluorescence staining to analyze the ovarian expression patterns of eight DEPs and found that they were expressed in granulosa cells and cumulus complexes (Figure 4D). This study constructed a Venn diagram for the DEPs using multiple comparisons of the endocrine, reproductive, and neural signal transduction system groups (Figure 4E). Thus, we screened three DEPs (FST, NR5A1 and PRL) and examined the expression patterns of three proteins in the hypothalamic–pituitary–ovarian axis and genital organs (oviduct and uterus).

### 3.5. Analysis of Tissue Expression

The FST, NR5A1, and PRL expression patterns were analyzed via PCR, Western blot, and immunofluorescence staining. *FST, NR5A1* and *PRL* mRNA were significantly differentially expressed in the hypothalamus, pituicytes, pineal gland, ovaries, oviducts, uterus, and other tissues.

**Figure 5:**
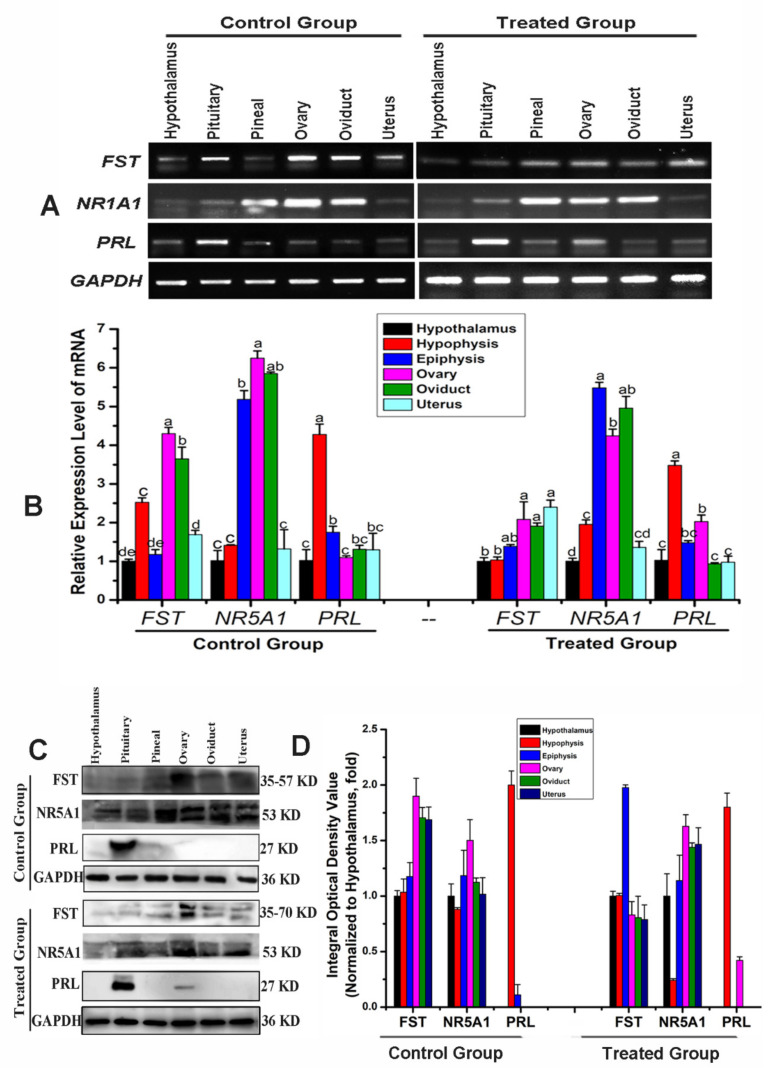
Expression and localization analysis of the differentially expressed proteins (DEPs) in the hypothalamic–pituitary–ovarian axis. (**A**) *FST, NR5A1* and *PRL* mRNA expression levels in Bactrian camel tissues detected via RT-PCR. (**B**) Optical density was analyzed via PCR and ImageJ. Different letters indicate significant differences within groups. (**C**) Western blot analysis of FST, NR5A1 and PRL proteins in different tissue samples. (**D**) Optical density value was analyzed via ImageJ.

**Figure 6:**
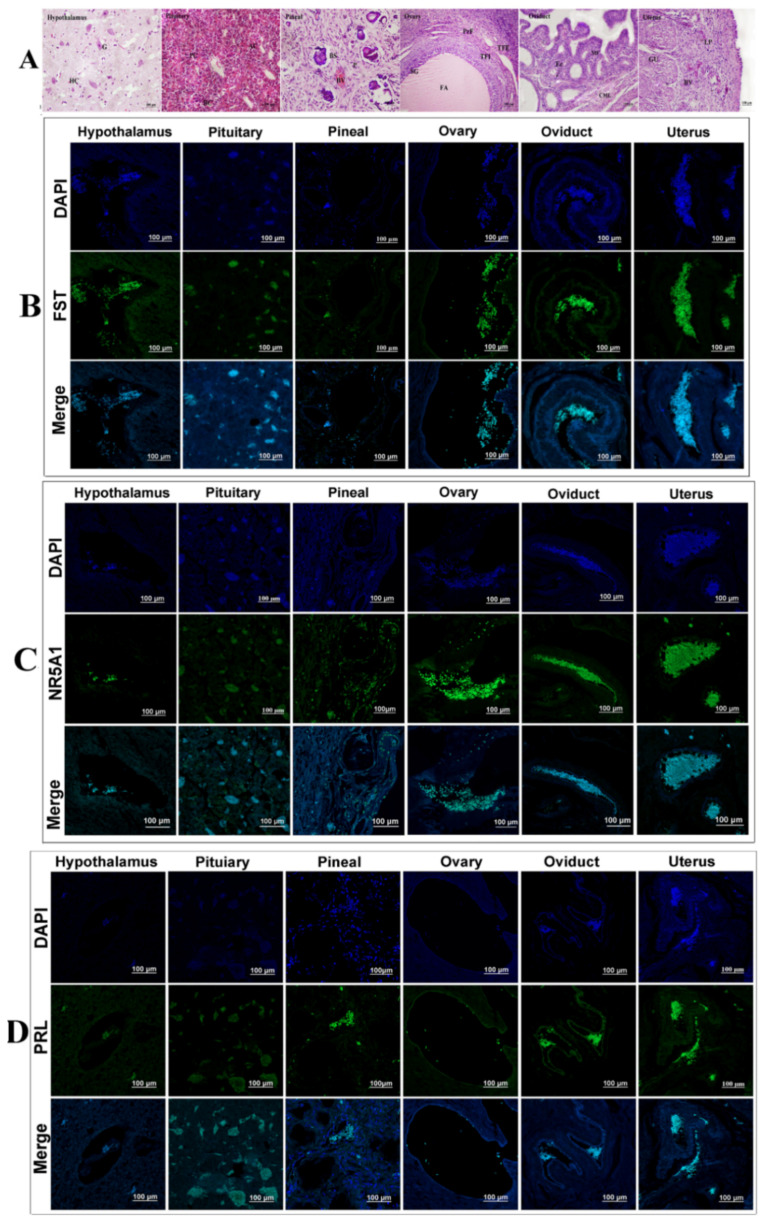
(**A**) Representative images of hematoxylin and eosin staining of the hypothalamus, pituitary, pineal gland, ovaries, oviduct, and uterus. (**B**) Intracellular localization analysis of FST in different tissues. (**C**) Intracellular localization analysis of NR5A1 in different tissues. (**D**) Intracellular localization analysis of PRL in different tissues.

In the control group, PCR results revealed that the ovaries and oviducts had the highest *FST* expression levels, with moderate expression in the pituitary and uterus and weak expression in the hypothalamus and pineal gland (Figure 5A,B). Immunoblotting results revealed that the ovaries and pituitary had the highest FST expressions, with moderate expression in the oviducts and uterus and weak expression in the hypothalamus and pineal gland (Figure 5C,D). In the treated group, PCR results revealed moderate expression in the pineal gland, ovaries, oviduct, and uterus and weak expression in the hypothalamus and pituitary (Figure 5A,B). Western blot results revealed strong FST expression in the hypothalamus, pituitary, pineal gland, ovaries, oviduct, and uterus. These expression patterns were opposite to those observed in the control group (Figure 5C,D). FST mRNA and protein expression levels in the hypothalamus, pituitary, pineal gland, ovaries, fallopian tubes, and uterus differed from those of the control group (Figure 6A–D). Immunofluorescence results suggested weak FST immunoreactivity in the hypothalamus and strong FST expression in the glia, pineal gland, pituicytes, ovarian granulosa cells, cumulus complexes, oviduct ciliated columnar epithelium, and secretory cells, as well as in the uterine, secretory, and epithelial cells of the endometrial gland (Figure 6A,B).

In the control group, *NR5A1* mRNA expression levels were highest in the ovaries, followed by the oviduct and pineal gland, and were weakest in the hypothalamus, pituitary, and uterus. Western blotting results revealed strong NR5A1 expression in the ovaries, oviduct, and uterus and moderate expression in the hypothalamus, pituitary, and pineal gland (Figure 5A,B). In the treated group, *NR5A1* mRNA expression was highest in the ovaries, oviduct, and pineal gland, with weak expression in other tissues. Immunoblotting results suggested that the protein expression trends differed from those of the transcription levels (Figure 5A,B). NR5A1 mRNA and protein expressions in the hypothalamus, pituitary, pineal gland, ovaries, oviduct, and uterus differed from those of the control group; however, the expression patterns were similar (Figure 5A–D). Immunofluorescence results revealed the expression levels for the cells, pineal cells, glia, pineal gland, pituicytes, ovarian granulosa cells, cumulus complexes, oviduct epithelial cells, and endometrial cells (Figure 5A,C).

In the control group, the pituitary contained the highest PRL mRNA and protein levels. The PCR results indicated weak *PRL* mRNA expression in the hypothalamus, ovary, oviduct, pineal gland, and uterus; however, Western blotting revealed strong PRL expression only in the pituitary (Figure 5A,B). In the treated group, the PCR results suggested that the pituitary had the highest *PRL* mRNA expression and that the ovary and pineal gland had the weakest *PRL* mRNA expression. Immunoblotting suggested a strong protein abundance in the pituitary and a weak protein abundance in the ovaries. No protein was detected in the hypothalamus, pineal gland, oviduct, or uterus (Figure 5C,D). The ovarian PRL mRNA and protein levels in the treatment group differed significantly, and the other tissues had similar expression patterns to those of the control group (Figure 5A–D). Immunofluorescence revealed different PRL expression levels in various tissues, with a weak PRL immunoreactivity in the blood vessels, nerve cells, glial cells, hypothalamus, pituitary gland cells, and nerve cells, as well as strong immunoreactivity in the pineal cells, variant granulosa cells, oviduct secretory epithelial cells, and endometrial cells (Figure 6A,D).

## 4. Discussion

Bactrian camels are induced ovulators, and their ovulatory mechanism appears to differ completely from those of spontaneous and other ovulators. During estrus, Bactrian camel follicles grow, develop and mature (diameter > 10 mm), and the female camels mate in order to ovulate. In a previous study, llamas that were administered intrauterine infusions of PBS or SP had follicles of 9.6 ± 0.8 mm or 8.8 ± 0.4 mm in diameter, respectively [20], A previous study on llamas found that LH peaked 2.5 h after normal male mating, and SP peaked at 4.5 h, then decreased progressively [21]. In alpaca SP, b-NGF was identified as one of the most abundant proteins via liquid chromatography-mass spectrometry [22]. Studies have shown that camel semen is rich in b-NGF, and this molecule is far more abundant in camel SP than in SP from other species (e.g., cows, horses, pigs, and sheep) [23]. Here, we induced ovulation using fresh SP in Bactrian camels, where the serum LH concentrations peaked at 4 h, then decreased progressively. Circulating E2 and PROG levels also increased and peaked at 12 h after mating. However, in female llamas that were administered SP, PROG reached its highest concentration after 7 days [24]. In the current study, serum FSH and PRL levels increased significantly and were higher in the treated group than in the control group. Serum GnRH peaked at 36 h, and its level was significantly higher than that of the control group. Within 0–48 h, the hormone levels in the control group were nearly stable. Ovulation in mammals involves GnRH secretion from the hypothalamus into the hypophyseal ovarian system, followed by the release of LH from the anterior pituitary into systemic circulation [25]. The ovulatory effect of OIFs in SP is mediated via a surge release of LH into the bloodstream. The Bactrian camel, the dromedary (*Camelus dromedarius*), the alpaca (*Lama pacos*), and the llama (*Lama glama*) are all induced ovulators. Chen B, X et al. suggested that one mature or developing follicle of Bactrian camels was usually present in an ovary. When not allowed to mate, the camel manifested prolonged periods of estrus; if mating occurred, then ovulation would take place 30–48 h later [11]; for Dromedaries, ovulation would take place at least 32–40 h later [26]; for llamas, then ovulation would take place 26 h later, however, if hCG and GnRH are injected, ovulation would take place 27.2 ± 0.3 h and 28.6 ± 0.3 h later [27,28]. A study to determine the site of action of OIFs found that treating llamas with a GnRH antagonist suppressed the effects of OIFs, suggesting a direct or indirect effect of OIFs on GnRH neurons in the hypothalamus [29].

Previous research on induced ovulation has mainly focused on isolating and purifying OIFs and hormone testing. We identified 5075 proteins after intramuscularly injecting SP, and found that 133 proteins were associated with reproduction and reproductive processes. We classified 404 DEPs (260 upregulated, 144 downregulated) via GO analysis and assigned them into three groups (i.e., molecular functions, biological process, and cellular component groups) using GO terms through multiple approaches. The upregulated and downregulated DEPs were then further clustered on the basis of their functions and signaling pathways, with a significant enrichment analysis. We also developed a PPI network complex of DEPs and identified 100 nodes/DEPs with 103 edges. Finally, qPCR and Western blot results revealed the expression profiles of ovulation-inducing and hormone-related genes and proteins, and immunofluorescence colocalization revealed that these DEPs were expressed in ovarian granulosa cells. Further screening of 69 DEPs related to neurochemical signal transduction, endocrine hormones, reproductive hormones, and PPI network mapping revealed 44 nodes and 77 edges. We further screened three proteins (FST, PRL, and NR5A1) using a Venn diagram. Finally, RT-PCR and Western blotting verified the expression profiles of three proteins in in the hypothalamic–pituitary–gonadal axis. Interestingly, the most significant module was filtered from the PPI network complex, and all three proteins were located in the PPI node. KEGG results indicated that FST, NR5A1, and PRL were involved in steroidogenic, oxytocin, follicular development, and oocyte meiosis processes.

Steroid-producing factor-1 (SF-1, NR5A1) is a member of the nuclear receptor superfamily and is an orphan nuclear receptor with a genetically distinct domain that is different from those of other nuclear receptors [30]. NR5A1 is a key transcriptional regulator of genes involved in the hypothalamic–pituitary–steroidogenic axis [31]. It is expressed in tissues such as the adrenal gland, gonad, hypothalamus, pituitary, skin, and spleen [32]. NR5A1 is highly expressed at steroid production sites. We found that NR5A1 was expressed in the fallopian tube epithelium, ovaries (granulosa cells and cumulus complex), hypothalamus, pituitary, pineal gland, and uterus of Bactrian camels. KEGG analysis suggested that NR5A1 is involved in steroidogenesis via cortisol synthesis/secretion pathways and the regulation of rate-limiting enzymes, suggesting that it may be involved in the secretion synthesis of E_2_, FSH, LH, PRL, and PROG. In mice, NR5A1 is expressed during the functional differentiation of steroidogenic tissues, and in the sexual differentiation of gonads [33]. Other research found that NR5A1 was expressed in the fetal and adult adrenal cortex, as well as in the testicular Leydig and Sertoli cells [34]. NR5A1 regulates the transcription of key genes involved in sexual development and reproduction, including steroidogenic acute regulatory protein, 17-alpha-hydroxylase, cytochrome P-450 cholesterol side-chain cleavage, and the beta subunit of LH [35]. NR5A1 is also expressed in nonsteroidogenic tissues, such as the ventromedial nucleus of the hypothalamus, pituitary gonadotrophs [36], placental venous blood vessels, sinus endothelial cells, skin, and the spleen [37]. In this study, camel NR5A1 was expressed at different levels in the tissues of the hypothalamic–pituitary–gonadal axis, the oviduct, and uterus. This study speculates that it may regulate steroid hormone synthesis and secretion.

Follistatin (FST) is a single-chain, secreted glycoprotein that can bind to many members of the transforming growth factor superfamily and inhibit their activity [38]. Earlier studies have found that FST is expressed in most body tissues and is highly expressed in fetuses and the heart, kidney, liver, ovaries, and pituitary [39,40]. Ovarian expression of FST in follicles and granulosa cells suggested that it may regulate granulosa cell differentiation through autocrine mechanisms [41]. This study found that FST was highly expressed in the fallopian tubes, ovaries, pituitary, and uterus and weakly expressed in the hypothalamus and pineal gland in Bactrian camels. We also found differences in FST expression levels between the treated and control groups. One study showed that loss of FST or Wnts resulted in the loss of mouse germ cells [42]. This study suggested that FST was mainly distributed within the glial cells of the hypothalamus and pituitary gland, ovarian granulosa cells, and uterine and fallopian tube cells.

PRL is a polypeptide hormone secreted mainly by the anterior pituitary. Carrasco et al. reported that OIFs and NGF mediated ovulation and lactation in camels and cattle [43]. This study found PRL in the ovaries and pituitary of the treated group. In the control group, PRL was strongly expressed only in the pituitary and was undetected in other tissues via immunoblotting. Immunofluorescence results showed different PRL expression levels in other tissues. PRL immunoreactivity has been found in numerous hypothalamic areas in other mammals, including rats [44]. Rats, mice, cows, pigs, and humans all have a placenta, amnion, decidua, and uterus [45]. Some well-established stimulators, such as ovarian steroids, modulate hypothalamic E2, angiotensin II, and vasoactive intestinal peptides and regulate hypothalamic and pituitary prolactin synthesis and secretion [46,47]. Studies have shown that hypothalamic prolactin-release inhibitors, prolactin-releasing factors, peripheral hormones, and PRL self-feedback regulate PRL secretion to promote gonadal development and reproductive behavior [48,49]. Estrogen also promotes PRL synthesis, storage, and release [50]. These results demonstrate the pivotal roles of FST, NR5A1, and PRL in modulating and activating the reproductive axis during induced ovulation in Bactrian camels.

## 5. Conclusions

In this study, we used RIA to measure hormones and B-ultrasound to detect follicular development, and identified ovarian DEPs in treated and control camels using iTRAQ labeling. This study identified 404 DEPs, and among them, FST, NR5A1, and PRL were involved in regulating induced ovulation and hormone secretion. The results verified the expression profiles of these three proteins via PCR, Western blot and immunofluorescence. Our study revealed distinct molecular functions and metabolic pathways that are active during Bactrian camel reproduction. These results demonstrated the pivotal roles of FST, NR5A1, and PRL in modulating and activating the reproductive axis during induced ovulation in Bactrian camels. Measurement of selected proteins using more targeted methods is a promising approach for studying the potential mechanisms of ovarian development and ovulation.

## Figures and Tables

**Table 1:** Primer sequences of the mouse genes used for qPCR.

Primer	Sequence 5′–3′	Product Length (bp)	Accession No.
*GAPDH*	gtggagccaagagggtcat	232	NW_011514262.1
	gggccatccacagtcttct		
*PRL*	gggcagagggttcatgact	176	XM_010974249.1
	taccccgcacttctgtgac		
*NR5A1*	tggtgttcgaccacatctacc	221	XM_010956204.1
	gaggctgaagaggatgaggaa		
*FST*	agacctgtcgggatgttttct	183	XM_010966300.1
	ggaggcaggtagcctttctta		

**Table 2:** Differentially expressed proteins in the ovarian tissues of Bactrian camels after SP-induced ovulation.

Different Expression	Protein Name
**UP**	CRT, GABRB2, HDGF, CLIC1, LOC10507404, PURH, PPP1CB, CLIC2, STX11, SAP30BP, SIPA1L1, PIP4K2C, ABCB9, CD63, TTC19, SOX1, EXOSC8, SAT2, CTR9, TMSB10, LDLR, F13A1, P3H4, LOC105068711, FGGY, TBC1D24, TMEM87A, Slc22A17, HSL, FOXK1, HSPB6, REXO2, Ighg1a, SEBOX, ATP1B1, MRPS26, CASK, Mrpl24, CCDC43, PHC2, UBE2N, MFF, LRRFIP1, NRIP2, COPE, SMARCB1, KRT8, CDHR2, C17ORF67, AGPS, HDAC3, CAMKK1, PON1, CTSS, GPX1, FKBP14, MRPL14, MTHFD2, CAPN2, THBS3, DDX19A, LGALSL, P3H1, JUND, BORCS6, SERPINH1, PTGES3, PTMS, TFCP2, TNFaIP2, NFYB, WAC, PTPN6, LAMTOR1, CDH1, LIPG, SPARC, BTF3, STMN1, MANF, UFM1, Igh, PRL, REX1BD, CNN3, NUDC, CNPY3, MBP, EDEM1, CHST14, PLCH1, PYCR1, STX2, SEC23B, CDC42EP5, GBP1, KRT39, LOC105082461, CRMP1, PCNP, CIAPIN1, HDDC3, B2M, MFSD10, PPL, GJA1, RARRES2, TFAP2B, SYVN1, FRAS1, MED1, PLP1, SMAP2, PSAT1, ELOB, QPCT, ATL2, SLC25A46, SCCA, GLT8D2, PSTPIP2, AGG, TGFB2, NDEL1, TCHH, BZW2, VPS13D, GSDMD, CRYZL1, CRABP2, CSTB, BCKDHB, RAB43, SPATA5, eEF2K, PRRC1, RER1, PSMF1, MRPL50, GABARAPL2, CTRL, SLC13A5, RABL6, MTRR, MRC2, NPC1, PLIN3, KRT7, PSME2, NAT10, COL6A5, CSN2, NSD1, SYT1, FKBP10, FDX1, TXN2, LGALS3, SLC35A3, HCLS1, MCRIP1, LOC105069208, SREK1, GLS, RPE, PPA1, LOC105070504, LRRC41, OTUB1, TSFM, TEAD1, LOC105073173, CDC123, IRF3, CALU, FABP5, KCNAB2, RWDD1, P4HA2, LOC105067606, SAR1B, MRPS25, LOC105069779, ERAP2, LOC105066647, LOC105067240, LBP, SDC4, TCN1, COPZ1, SAR1A, SLC46A1, JPT2, KRT18, VSNL1, DDX59, LOC105079327, CD109, CNN2, MAN2A1, ICOSLG, LOC105077404, PPP1R2, SLC1A5, S100A6, SEC11C, CTSV, RCN3, UBR2, PSMB10, TPT1, LOC105075883, IRF2BP2, LOC105074738, ARMCX3, ESPL1, HP, C1QTNF3, BABAM1, ELOC, GOLIM4, MRTFB, CDV3, ARHGAP32, TMEM94, TP53I3, GFPT2, CEP131, TYMS, PLPP3, PAK1, PPCA, LETMD1, GFAP, LRRC8D, MCF2L, ITIH4, SIGLEC1, SEC13, EI24, P3H3, CHMP5, POLR2I, WARS1, SELENOH, aLG11, ITIH3, MRFAP1, CEP104, MRPS31, DHCR24, MRPS36, PLA2G6, VTA1, HYPK, STAG2.
**Down**	ELMO2, LAP2, NAT14, ESPN, LPAAT1, DT S7, NEFL, Wnt2b, MCAM, Rint1, ARF5, BRPF1, WASH1, c-Rel, TPD52, KRT19, TSTD3, CDK19, PTPP, EXOC6, CRT11, TMEM209, LOC105080173, ISPS, Di-Ras1, RIM11, FST, GGTB, P400, TESTIN, PRELP, LMOD1, TINAGL1, TNFAIP8L3, HOXD8, SCO2, ZCCHC3, Septin4, NIPSNAP1, NUMB, ZBED5, DHX35, PATZ, ALDH3B1, LAMA4, RPL11, NR5A1, PDX1, CLYBL, MYH11, ABCC8, VPS4A, GLRX1, LIMS2, PPP1R10, LOC105068711, KRT1, Ybx3, MVI, ANAPC7, LAMB2, MYZAP, MED1, ZNF410, PFDN6, Smoothelin, OR, PYCR2, MSH3, PNPLA7, CAMK2G, KRT17, PPBP, DDX31, MRPL19, ZER1, MSTO1, KCR1, RPS27L, RyR3, QXPO4, Desmin, FBXO7, HSPA2, PGPEP1, MYO7, Lamc1, A4D1P6, NOB1, ZNF326, TTC21B, CBL, RMC1, TRIM32, LIN9, SHPK, EPC1, PBX2, SVEP1, DCTN4, PRKCD, HEBP2, EML6, DDT, LENG8, RPRD2, ECHDC3, PLIN4, H2A, BAG2, FLNA, HMCN2, PLEKHH2, OSTC, CAVIN3, AHSP, GTPBP3, ARID4B, ART4, TINF2, CTU2, MEN1, RNF123, MARK1, CCDC127.

## Data Availability

The mass spectrometry proteomics data have been deposited to the ProteomeXchange Consortium (http://proteomecentral.proteomexchange.org, accessed on 26 November 2019) via the iProX partner repository with the dataset identifier PXD016896 (https://www.iprox.org/page/project.html?id=IPX0001930000, accessed on 27 November 2019), For quantitative and analytical data, see Tables S1 and S2.

## Supplementary Materials

The following are available online at https://www.mdpi.com/article/10.3390/ani11123512/s1, Figure S1. (A) B-ultrasound detected the development of follicles from the control group. (B) B-ultrasound detected the development of follicles from the treated group. Table S1. Bactrian camels SP-induced ovulation up-regulated differential protein list of ovarian tissues; Table S2. Bactrian camel SP-induced ovulation down-regulated differential protein list of ovarian tissues.
