# Supplementary material for: Screening and Identification of Differential Ovarian Proteins before and after Induced Ovulation via Seminal Plasma in Bactrian Camels
Source: Animals (Basel). 2021 Dec 9;11(12):3512. doi: 10.3390/ani11123512 (PMC8698062; doi:10.3390/ani11123512)
Supplement: Supplementary file 1 [file animals-11-03512-s001.zip › Figure S3.pdf]

1: Hypothalamus 2: Pituitary 3: Pineal 4: Ovary 5: Oviduct 6: Uterus

Control group

1 2 3 4 5 6

2000

FST
183bp

NR5A1
2251bp

PRL
176bp

GAPDH
232bp

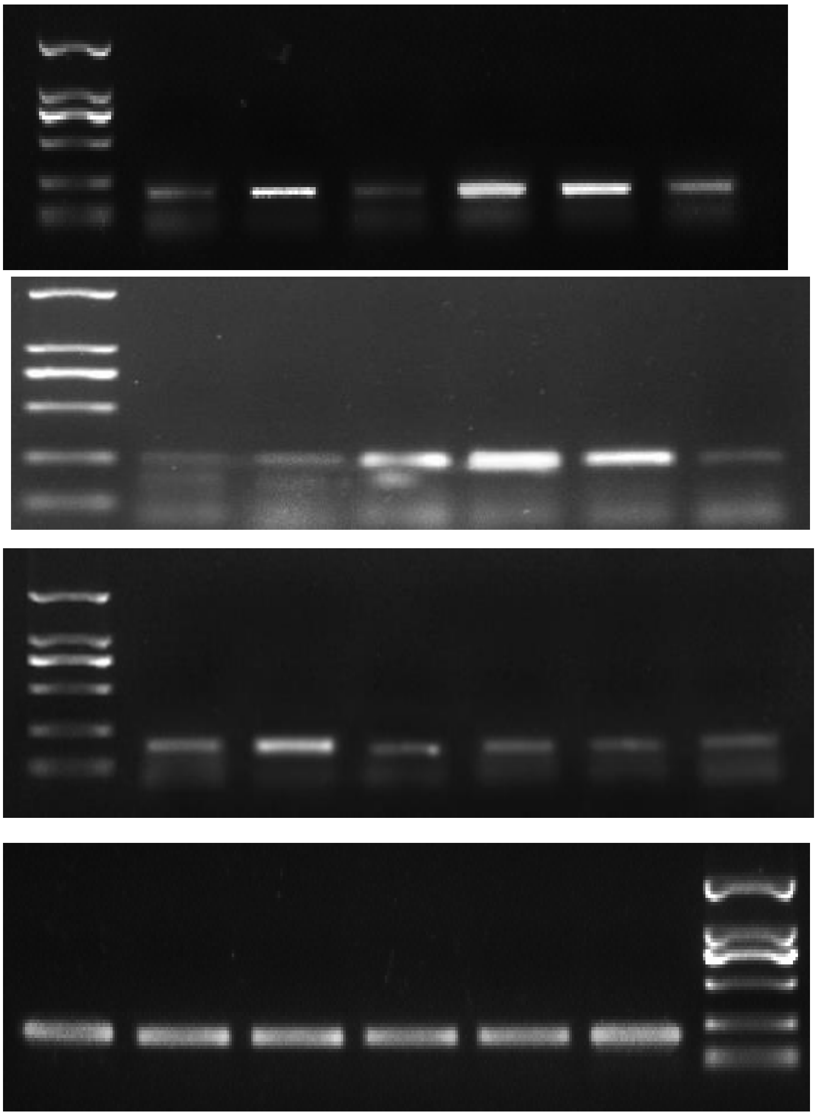

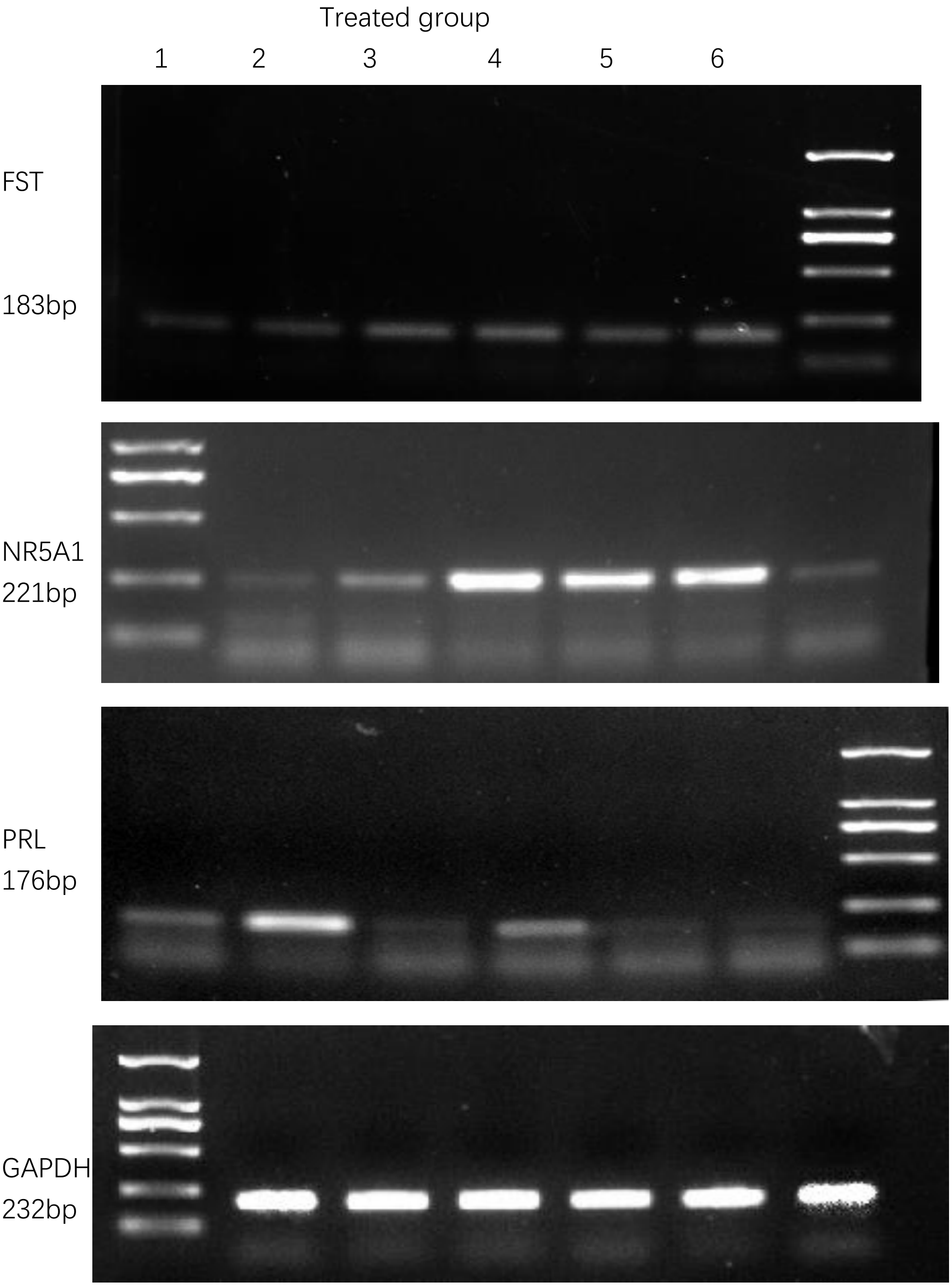
Treated group
1
2
3
4
5
6
FST
183bp
NR5A1
221bp
PRL
176bp
GAPDH
232bp

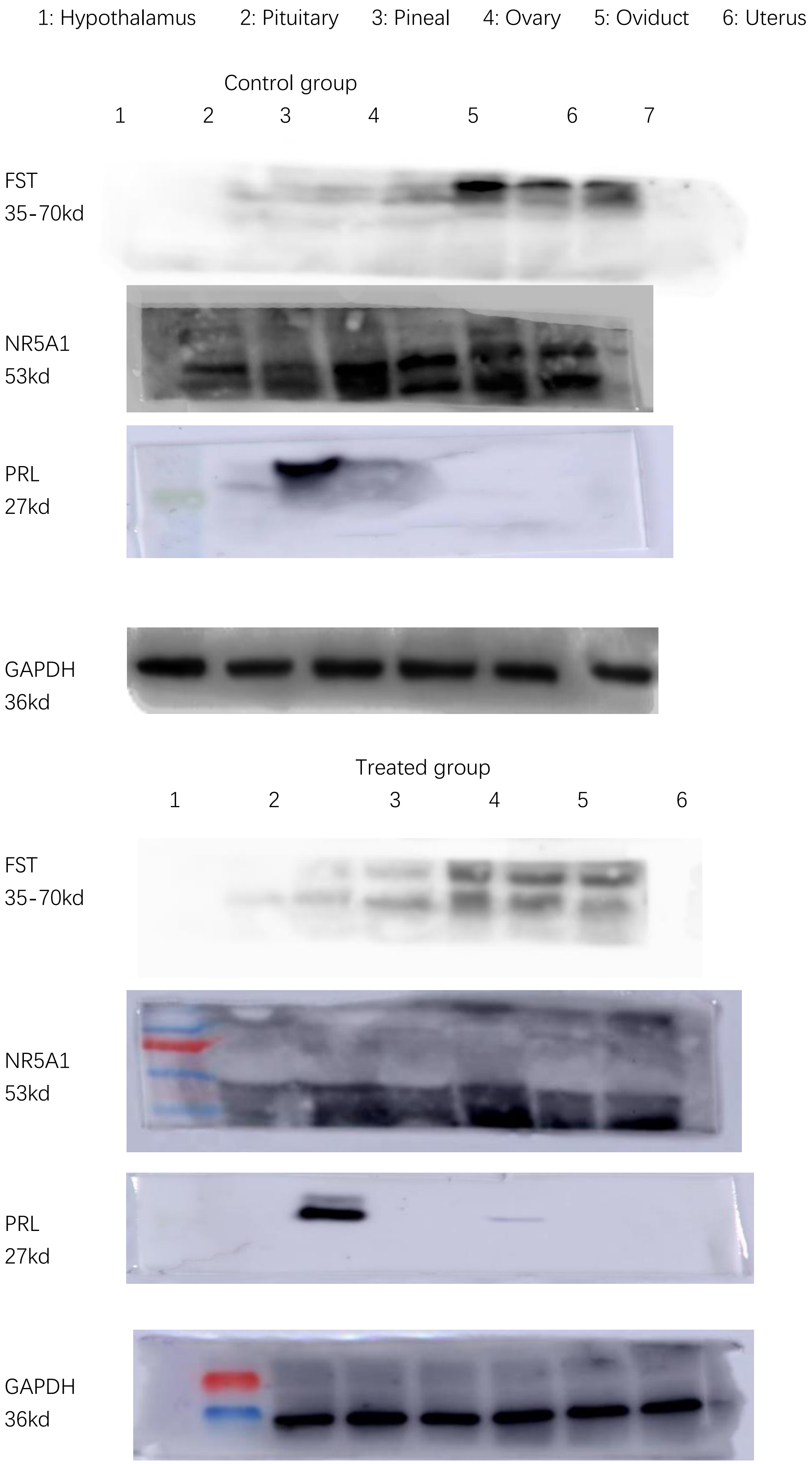
1: Hypothalamus 2: Pituitary 3: Pineal 4: Ovary 5: Oviduct 6: Uterus
Control group
1 2 3 4 5 6 7
FST
35-70kd
NR5A1
53kd
PRL
27kd
GAPDH
36kd
Treated group
1 2 3 4 5 6
FST
35-70kd
NR5A1
53kd
PRL
27kd
GAPDH
36kd
