# Supplementary material for: Screening and Identification of Differential Ovarian Proteins before and after Induced Ovulation via Seminal Plasma in Bactrian Camels
Source: Animals (Basel). 2021 Dec 9;11(12):3512. doi: 10.3390/ani11123512 (PMC8698062; doi:10.3390/ani11123512)
Supplement: Supplementary file 1 [file animals-11-03512-s001.zip › Table S1.pdf]

Table S1 Bactrian camel SP-induced ovulation up-regulated differential protein list of ovarian tissues

Protein_ID	GENE	Mass	Protein_Coverage	Uniq_Pep_Num	Uniq_Spec_Num	Mean_Ratio_treated-VS-control	SD_treated-VS-control	Quant_Num_treated-VS-control	Pvalue_treated-VS-control
XP_010970587.1	CRT	50618.12	0.016	1	3	1.37	0.452	9	0.04021
XP_010970524.1	GABRB2	54735.33	0.065	1	1	1.27	0.254	9	0.01544
XP_010965236.1	HDGF	67972.16	0.04	1	1	1.43	0.542	9	0.04276
XP_010959797.1	CLIC1	26096.42	0.287	5	12	1.24	0.21	9	0.007629
XP_010959778.1	LOC10507404	84271.21	0.164	1	2	1.3	0.33	9	0.03483
XP_010958351.1	PURH	65191.54	0.149	7	10	1.25	0.123	9	0.0001512
XP_010967942.1	PPP1CB	37682.99	0.229	1	1	1.24	0.302	9	0.04389
XP_010965398.1	CLIC2	28483.45	0.308	7	10	1.27	0.142	9	0.0001719
XP_010963975.1	STX11	33911.94	0.028	1	1	1.24	0.135	9	0.0003401
XP_010949058.1	SAP30BP	33954.73	0.039	1	1	1.22	0.25	9	0.0267
AEA40696.1	SIPA1L1	28717.41	0.027	1	1	4.75	2.579	9	4.18E-05
XP_010944620.1	PIP4K2C	47206.05	0.017	1	1	1.34	0.284	9	0.004183
XP_010968006.1	ABCB9	84884.92	0.009	1	2	1.26	0.231	9	0.008002
XP_010962205.1	CD63	26416.49	0.089	2	2	1.42	0.379	9	0.007959
XP_010972371.1	TTC19	30181.37	0.046	1	1	1.3	0.252	9	0.005365
XP_010953226.1	SOX1	79312	0.091	5	12	1.29	0.345	9	0.04564
XP_010949787.1	EXOSC8	30629.49	0.058	1	1	1.56	0.494	9	0.003817
XP_010964223.1	SAT2	19398.8	0.265	4	5	1.39	0.29	9	0.002423
XP_010963171.1	CTR9	134306.6	0.015	1	1	1.54	0.431	9	0.003426
XP_010957214.1	TMSB10	4759.441	0.143	1	1	1.48	0.227	9	0.0001116
XP_010966219.1	LDLR	95750.89	0.008	1	1	1.48	0.195	9	2.25E-05
XP_010966898.1	F13A1	83540.81	0.277	19	38	1.25	0.269	9	0.02301

XP_010957790.1	P3H4	39436.94	0.024	1	1	1.33	0.397	9	0.02967
XP_010971053.1	C20orf27	20147.05	0.078	1	2	1.72	0.983	9	0.04536
XP_010966834.1	FGGY	50313.13	0.4	16	54	1.26	0.298	9	0.04608
XP_010948166.1	TBC1D24?	62351.57	0.044	2	2	1.21	0.256	9	0.03072
XP_010963492.1	TMEM87A	63843.25	0.016	1	1	1.34	0.179	9	0.0002301
XP_010949545.1	Slc22A17?	37781.42	0.072	2	3	1.73	0.758	9	0.02504
XP_010945022.1	HSL	86588.04	0.013	1	1	1.4	0.27	9	0.001707
XP_010964529.1	FOXK1	77605.96	0.025	1	1	1.47	0.281	9	0.0004694
XP_010948414.1	HSPB6	17939.46	0.143	2	9	1.21	0.225	9	0.02966
CCF72131.1		24837.52	0.735	2	27	1.3	0.375	9	0.03866
XP_010959773.1	Ighg1a	196437.6	0.258	41	112	1.39	0.31	9	0.00341
XP_010956643.1	SEBOX	27249.8	0.044	1	10	1.31	0.352	9	0.02311
XP_010952134.1	ATP1B1	35392	0.152	5	13	1.23	0.206	9	0.008245
XP_010971077.1	MRPS26	23788.68	0.054	1	1	1.54	0.61	9	0.02178
XP_010952100.1	CASK	21977.32	0.136	3	5	1.24	0.289	9	0.03849
XP_010952176.1	mrpl24.L	24990.08	0.065	1	1	1.24	0.195	9	0.003976
XP_010957882.1	CCDC43	23088.98	0.055	1	1	1.22	0.244	9	0.02385
XP_010950435.1	PHC2	85805.61	0.042	3	3	1.27	0.126	9	9.65E-05
XP_010961018.1	UBE2N	16290.37	0.347	6	19	1.21	0.118	9	0.0004771
XP_010946440.1	LRRFIP1	88362.05	0.05	1	1	1.65	0.692	9	0.0165
XP_010962816.1	NRIP2	26466.45	0.163	3	5	1.36	0.193	9	0.0002676
XP_010970479.1	COPE	34589.18	0.097	3	4	1.21	0.08	9	3.36E-05
XP_010953759.1	SMARCB1	43397.68	0.061	2	3	1.72	0.287	9	1.48E-05
XP_010962653.1	KRT8	53346.17	0.342	14	39	1.61	0.653	9	0.02604
XP_010944511.1	CDHR2	142350.8	0.006	1	1	1.34	0.314	9	0.01032
XP_010948925.1	C17ORF67	20888.84	0.091	2	2	1.81	0.802	9	0.02769

XP_010958259.1	AGPS	64582.4	0.051	2	2	1.21	0.159	9	0.00249
XP_010948843.1	HDAC3	49482.68	0.054	2	2	1.28	0.185	9	0.001476
XP_010968245.1	CAMKK1	56229.8	0.061	3	3	1.23	0.104	9	8.50E-05
XP_010970850.1	PON1	40067.73	0.228	7	10	1.45	0.52	9	0.02033
XP_010947191.1	MFF	37412.33	0.034	1	1	1.22	0.249	9	0.02343
XP_010956773.1	CTSS	38479.6	0.056	2	4	1.24	0.196	9	0.004534
XP_010967560.1	GPX1	19917.08	0.48	8	24	1.24	0.159	9	0.001662
XP_010969719.1	FKBP14	24330.48	0.071	1	1	1.24	0.192	9	0.004099
XP_010972118.1	MRPL14	15979.52	0.048	1	1	2.09	1.476	9	0.03994
XP_010950742.1	MTHFD2	38397.51	0.065	2	3	1.34	0.219	9	0.001246
XP_010972276.1	CAPN2	80713.98	0.259	17	47	1.21	0.238	9	0.03049
XP_010952251.1	THBS3	104260.2	0.007	1	1	1.29	0.267	9	0.01042
XP_010959315.1	DDX19A	56113.95	0.181	1	1	1.26	0.135	9	0.0002425
XP_010950224.1	LGALSL	19167.73	0.116	2	2	1.26	0.283	9	0.02453
XP_010964311.1	BORCS6	30658.51	0.052	1	1	1.28	0.21	9	0.002125
XP_010970454.1	JUND	35159.22	0.04	1	1	1.41	0.446	9	0.0246
XP_010971452.1	SERPINH1	46569.11	0.481	18	73	1.26	0.163	9	0.000875
XP_010962125.1	PTGES3	18952.52	0.344	6	14	1.31	0.3	9	0.01339
XP_010956138.1	PTMS	28892.46	0.041	1	2	1.27	0.257	9	0.01098
XP_010962599.1	TFCP2	57232.69	0.048	2	2	1.22	0.154	9	0.002125
XP_010967101.1	LOC105079952	24879.02	0.039	1	1	1.29	0.371	9	0.03955
XP_010968752.1	NFYB	22192.59	0.065	1	1	1.24	0.281	9	0.02849
XP_010956665.1	WAC	65587.72	0.01	1	1	1.26	0.183	9	0.002608
XP_010956135.1	PTPN6	71124.59	0.019	1	1	1.22	0.278	9	0.04437
XP_010971369.1	LAMTOR1	17815.85	0.155	2	2	1.3	0.277	9	0.00994
XP_010960833.1	CDH1	88023.55	0.018	1	1	1.26	0.342	9	0.04788

XP_010959691.1	LIPG	46454.2	0.061	2	2	1.35	0.266	9	0.00423
XP_010968315.1	SPARC	35311.93	0.109	4	8	1.51	0.236	9	4.76E-05
XP_010947600.1	BTF3	22232.47	0.034	1	1	1.22	0.279	9	0.04659
XP_010946345.1	STMN1	17273.94	0.181	3	14	1.32	0.378	9	0.03259
XP_010967414.1	MANF	20707.76	0.335	6	8	1.27	0.149	9	0.0003941
XP_010949773.1	UFM1	9150.884	0.259	2	4	1.24	0.187	9	0.003457
ALN98712.1	Igh-V	13124.24	0.075	1	1	1.36	0.428	9	0.04396
XP_010972551.1	PRL	26483.38	0.114	2	4	2.38	1.815	9	0.02374
XP_010970473.1	REX1BD	18074.19	0.062	1	1	1.24	0.175	9	0.001971
XP_010948723.1	CNN3	31891.64	0.299	6	12	1.21	0.255	9	0.02887
XP_010946298.1	NUDC	38209.14	0.343	10	16	1.21	0.213	9	0.01394
XP_010972062.1	CNPY3	31870.3	0.152	5	6	1.23	0.145	9	0.000845
XP_010954422.1	MBP	33380.44	0.088	2	3	2.06	1.313	9	0.02113
XP_010953661.1	EDEM1	18171.04	0.07	1	1	1.26	0.267	9	0.02129
XP_010963444.1	CHST14	39116.1	0.036	1	1	1.85	0.79	9	0.004381
XP_010950415.1	PLCH1	186559.4	0.005	1	4	1.21	0.235	9	0.02612
XP_010948947.1	PYCR1	33750.78	0.297	4	9	1.22	0.2	9	0.01109
XP_010946669.1	STX2	29904.6	0.031	1	1	1.23	0.107	9	0.0001097
XP_010971218.1	SEC23B	87621.6	0.029	1	1	1.85	0.45	9	8.23E-05
XP_010960362.1	CDC42EP5	11867	0.114	1	1	1.26	0.16	9	0.0007525
XP_010948669.1	GBP1	71173.32	0.134	9	9	1.28	0.222	9	0.002889
XP_010958733.1	KRT39	57076.58	0.012	1	1	1.67	0.373	9	0.0002447
XP_010958064.1	P3H1	64554.36	0.071	4	5	1.21	0.166	9	0.003999
XP_010970326.1	LOC105082461	18581.5	0.051	1	1	1.35	0.276	9	0.003223
XP_010971279.1	CRMP1	55522.04	0.081	1	1	1.34	0.217	9	0.0007596
XP_010964678.1	PCNP	18919.43	0.185	3	4	1.63	0.429	9	0.001267

XP_010965598.1	CIAPIN1	33752.95	0.042	1	2	1.28	0.321	9	0.03823
XP_010953396.1	HDDC3	20263.63	0.179	2	2	1.57	0.593	9	0.02875
XP_010963553.1	B2M	13743.05	0.136	2	4	1.22	0.212	9	0.01321
XP_010957502.1	MFSD10	48796.27	0.024	1	1	1.23	0.154	9	0.001409
XP_010948079.1	PPL	201264.9	0.095	13	13	1.22	0.201	9	0.008372
XP_010963792.1	GJA1	43037.77	0.037	1	1	1.61	0.625	9	0.02303
XP_010953140.1	RARRES2	14971.54	0.084	1	1	1.53	0.471	9	0.005335
XP_010972178.1	TFAP2B	48559.44	0.016	1	1	1.89	1.131	9	0.04143
XP_010955419.1	SYVN1	64187.67	0.016	1	1	1.41	0.322	9	0.004444
XP_010967704.1	FRAS1	452188.4	0.003	1	1	3.37	1.369	9	1.86E-05
ALB75438.1		37282.64	0.682	1	13	1.36	0.407	9	0.02687
XP_010966098.1	PLP1	26922.44	0.033	1	2	2.36	1.821	9	0.03487
XP_010958106.1	SMAP2	46929.84	0.03	1	1	1.24	0.217	9	0.006828
XP_010951092.1	PSAT1	40777.83	0.024	1	1	1.3	0.26	9	0.008222
XP_010948158.1	ELOB	13216.65	0.449	4	6	1.23	0.255	9	0.02942
XP_010965962.1	QPCT	40991.05	0.017	1	1	1.28	0.332	9	0.03767
XP_010965968.1	ATL2	64417.06	0.039	2	3	1.35	0.308	9	0.009766
XP_010969779.1	SLC25A46	46856.34	0.029	1	1	1.25	0.254	9	0.01783
XP_010967048.1	SCCA	47440.58	0.267	5	50	1.47	0.39	9	0.00577
XP_010968747.1	GLT8D2	40258.74	0.029	1	1	1.39	0.281	9	0.001928
XP_010959744.1	PSTPIP2	42464.4	0.071	1	1	1.3	0.259	9	0.006604
XP_010955507.1	AGG	23212.41	0.348	8	69	2.48	1.104	9	0.0004781
XP_010972315.1	TGFB2	41238.88	0.022	1	1	1.31	0.293	9	0.01208
XP_010964266.1	NDEL1	38558.46	0.035	1	1	1.27	0.234	9	0.005279
XP_010956698.1	TCHH	143783.1	0.011	1	1	1.76	1.033	9	0.0422
XP_010965456.1	BZW2	48250.06	0.017	1	1	1.21	0.175	9	0.006211

XP_010955819.1	VPS13D	491151	0.001	1	1	1.78	0.845	9	0.01129
XP_010959452.1	GSDMD	46664.7	0.028	1	1	1.27	0.303	9	0.02304
XP_010953203.1	CRYZL1	39237.26	0.113	3	3	1.24	0.175	9	0.002824
XP_010952181.1	CRABP2	15892.11	0.065	1	1	1.47	0.322	9	0.001555
XP_010964825.1	CSTB	11133.61	0.357	3	7	1.23	0.102	9	6.81E-05
XP_010971148.1	BCKDHB	43830.17	0.031	1	1	1.34	0.439	9	0.04292
XP_010959541.1	RAB43	23321.68	0.038	1	1	1.21	0.161	9	0.002996
XP_010950874.1	SPATA5	97316.57	0.014	1	1	1.24	0.235	9	0.009821
XP_010959425.1	eEF2K	82168.81	0.033	2	2	1.38	0.181	9	0.0001058
XP_010969866.1	PRRC1	46263.77	0.099	4	7	1.31	0.092	9	3.62E-06
XP_010955944.1	RER1	23006.81	0.041	1	1	1.52	0.491	9	0.01335
AOR39887.1		7889.251	0.149	1	1	1.28	0.355	9	0.04312
XP_010950539.1	MRPL50	18173.35	0.075	1	1	1.23	0.143	9	0.0007823
XP_010965634.1	GABARAPL2	13697.08	0.291	3	4	1.25	0.316	9	0.03331
XP_010960867.1	CTRL	26709.87	0.098	1	1	1.33	0.155	9	8.13E-05
XP_010952968.1	SLC13A5	58524.37	0.01	1	1	1.26	0.199	9	0.002768
XP_010952768.1	RABL6	75992.72	0.087	1	1	1.27	0.114	9	4.82E-05
XP_010968679.1	MTRR	78048.01	0.016	1	1	1.22	0.193	9	0.01166
XP_010954309.1	MRC2	169098.8	0.058	6	8	1.23	0.228	9	0.01373
XP_010956348.1	NPC1	131866.1	0.01	1	2	1.21	0.148	9	0.001856
XP_010965045.1	PLIN3	51172.67	0.104	5	6	1.23	0.205	9	0.007675
XP_010962632.1	KRT7	49030.27	0.181	7	17	1.26	0.282	9	0.02357
XP_010953056.1	PSME2	27501.36	0.079	3	3	1.24	0.224	9	0.01187
XP_010962997.1	NAT10	116832.2	0.012	1	1	1.37	0.399	9	0.03543
XP_010959542.1	COL6A5	249545	0.061	11	16	1.45	0.473	9	0.0123
CDO50355.1	CSN2	26179.05	0.06	1	1	1.35	0.287	9	0.003164

XP_010944502.1	NSD1	268921	0.005	1	1	1.85	1.145	9	0.04502
XP_010957133.1	SYT1	47611.72	0.026	1	1	1.54	0.638	9	0.02444
XP_010966342.1	FKBP10	58102.8	0.234	9	12	1.38	0.147	9	1.65E-05
XP_010967568.1	FDX1	18018.85	0.048	1	1	1.28	0.125	9	7.45E-05
XP_010960491.1	LGALS3	27644.69	0.255	7	22	1.24	0.1	9	5.14E-05
XP_010958675.1	TXN2	18462.65	0.09	1	2	1.29	0.262	9	0.0112
XP_010948742.1	SLC35A3	25696.54	0.026	1	1	1.25	0.158	9	0.0009091
XP_010969177.1	HCLS1	54394.89	0.02	1	1	1.22	0.231	9	0.01966
XP_010948959.1	MCRIP1	11017.58	0.113	1	1	1.41	0.397	9	0.008223
XP_010953588.1	LOC105069208	42190.97	0.103	3	5	1.24	0.16	9	0.00132
XP_010947658.1	SREK1	71920.25	0.022	1	1	1.24	0.234	9	0.01504
XP_010947015.1	GLS	47517.48	0.083	1	1	1.29	0.127	9	6.53E-05
XP_010958375.1	RPE	17710.77	0.094	2	3	1.23	0.19	9	0.005394
XP_010949953.1	PPA1	33232.37	0.066	2	3	1.45	0.256	9	0.000266
XP_010955224.1	LOC105070504	11302.72	0.136	2	9	1.64	0.716	9	0.02511
XP_010949165.1	LRRC41	83789.22	0.009	1	1	1.39	0.488	9	0.04652
XP_010955350.1	OTUB1	31505.5	0.177	4	14	1.23	0.249	9	0.02037
XP_010944645.1	TSFM	35681.28	0.12	3	5	1.21	0.171	9	0.005102
XP_010963156.1	TEAD1	46602.51	0.024	1	2	1.41	0.371	9	0.008381
XP_010958680.1	LOC105073173	37623.05	0.05	2	2	1.21	0.114	9	0.0003458
XP_010952017.1	CDC123	39417.46	0.042	1	1	1.29	0.223	9	0.003088
XP_010945290.1	IRF3	52190.02	0.034	2	2	1.21	0.164	9	0.003154
XP_010945864.1	CALU	37119.55	0.187	5	5	1.33	0.176	9	0.0001598
XP_010950021.1	FABP5	15380.63	0.37	4	8	1.32	0.202	9	0.0007893
XP_010955912.1	KCNAB2	33830.42	0.033	1	1	1.23	0.198	9	0.006942
XP_010963763.1	RWDD1	27933.42	0.037	1	2	1.29	0.288	9	0.014

XP_010969883.1	P4HA2	60960.7	0.053	2	2	1.32	0.069	9	2.61E-07
XP_010951498.1	GNAQ	53877.54	0.086	1	1	1.27	0.327	9	0.04282
XP_010969931.1	SAR1B	22477.52	0.197	1	2	1.32	0.204	9	0.0009541
XP_010951402.1	MRPS25	20390.5	0.098	1	1	1.51	0.429	9	0.003341
XP_010954283.1	LOC105069779	21670.01	0.16	3	4	1.22	0.232	9	0.01892
XP_010947842.1	ERAP2	110101.3	0.058	5	5	1.32	0.275	9	0.008698
XP_010950347.1	LOC105066647	64425.06	0.025	1	1	1.27	0.338	9	0.03834
XP_010951015.1	LOC105067240	23480.15	0.169	3	4	1.39	0.327	9	0.005236
XP_010959040.1	LBP	48092.16	0.184	7	11	1.66	0.334	9	0.0001347
XP_010956986.1	SDC4	21706.07	0.045	1	1	1.36	0.109	9	3.48E-06
XP_010954664.1	TCN1	43265.48	0.088	3	3	1.33	0.22	9	0.001172
XP_010962724.1	COPZ1	20224.48	0.226	3	4	1.22	0.114	9	0.0001844
XP_010949955.1	SAR1A	22490.55	0.197	1	1	1.44	0.512	9	0.03426
XP_010956587.1	SLC46A1	50851.35	0.028	1	1	1.8	0.799	9	0.01027
XP_010948214.1	JPT2	19717.83	0.261	3	3	1.25	0.272	9	0.02229
XP_010962654.1	KRT18	48024.29	0.301	11	18	1.42	0.378	9	0.01121
XP_010945655.1	VSNL1	22281.02	0.094	2	2	1.33	0.385	9	0.04225
XP_010954570.1	DDX59	67708.48	0.019	1	1	1.23	0.269	9	0.02762
XP_010966333.1	LOC105079327	46721.12	0.27	7	16	1.63	0.527	9	0.003734
XP_010971176.1	CD109	163905.5	0.116	14	19	1.28	0.123	9	8.94E-05
XP_010964927.1	CNN2	33727.36	0.107	3	8	1.26	0.171	9	0.001127
XP_010969775.1	MAN2A1	132119.4	0.044	5	5	1.22	0.073	9	1.31E-05
XP_010964862.1	ICOSLG	35239.51	0.032	1	1	1.61	0.44	9	0.001929
XP_010964108.1	LOC105077404	20475.89	0.073	1	1	1.3	0.264	9	0.006788
XP_010957592.1	PPP1R2	22811.91	0.131	2	4	1.27	0.241	9	0.008378
XP_010945160.1	SLC1A5?	56882.07	0.032	1	1	1.21	0.131	9	0.0007703

XP_010952355.1	S100A6	10150.27	0.278	3	4	1.39	0.224	9	0.0004951
XP_010959596.1	SEC11C	17337.23	0.11	2	2	1.28	0.235	9	0.00619
XP_010965767.1	CTSV	38175.14	0.021	1	2	1.21	0.21	9	0.01596
XP_010945282.1	RCN3	37533.11	0.186	5	17	1.29	0.131	9	0.000116
XP_010972239.1	UBR2	204809.7	0.014	2	3	1.25	0.223	9	0.007311
XP_010960804.1	PSMB10	29351.04	0.026	1	1	1.21	0.223	9	0.01783
XP_010949687.1	TPT1	19664.6	0.267	4	16	1.28	0.212	9	0.003027
XP_010962145.1	LOC105075883	28300.77	0.13	3	9	1.69	0.627	9	0.003506
XP_010961148.1	IRF2BP2	39028.45	0.058	1	1	1.31	0.326	9	0.01792
XP_010960674.1	LOC105074738	53726.34	0.026	1	1	1.36	0.362	9	0.01697
XP_010968228.1	ARMCX3	41451.32	0.036	1	1	1.42	0.396	9	0.009178
XP_010962662.1	ESPL1	233503.3	0.003	1	15	1.42	0.537	9	0.04822
XP_010958959.1	HP?	39028.83	0.36	15	47	2.98	1.144	9	3.15E-05
XP_010953856.1	C1QTNF3	33814.37	0.033	1	1	1.39	0.324	9	0.003144
XP_010970417.1	BABAM1	37284.86	0.154	4	6	1.35	0.238	9	0.00145
XP_010949743.1	ELOC	12618.05	0.357	3	5	1.3	0.225	9	0.003324
XP_010950383.1	GOLIM4	79112.13	0.035	2	2	1.21	0.231	9	0.02755
XP_010960028.1	MRTFB	117389	0.009	1	1	1.39	0.206	9	0.0002498
XP_010946027.1	CDV3	22147.67	0.037	1	1	1.84	1	9	0.02458
XP_010956179.1	ARHGAP32	208272.8	0.007	1	1	2.56	1.768	9	0.02246
XP_010949070.1	TMEM94	154100	0.015	2	2	1.52	0.492	9	0.01534
XP_010946881.1	TP53I3	35724.72	0.045	1	2	1.34	0.319	9	0.007436
XP_010944441.1	GFPT2	73435.84	0.067	2	3	1.3	0.184	9	0.0006041
XP_010949255.1	CEP131	122578.2	0.007	1	1	1.21	0.248	9	0.03265
XP_010956369.1	TYMS	29223.74	0.043	1	1	1.64	0.63	9	0.008597
XP_010956666.1	PLPP3	35689.26	0.061	2	2	1.27	0.169	9	0.0007928

XP_010971816.1	PAK1	58951.88	0.093	1	1	1.23	0.235	9	0.01707
XP_010956960.1	PPCA	54716.03	0.082	2	3	1.23	0.282	9	0.03327
XP_010962596.1	LETMD1	42258.51	0.025	1	1	1.31	0.243	9	0.002403
XP_010957808.1	GFAP	46628.73	0.09	2	2	1.78	0.909	9	0.02298
XP_010948675.1	LRRC8D	98658.24	0.029	1	1	1.43	0.357	9	0.003159
XP_010950635.1	MCF2L	124700.4	0.01	1	1	1.41	0.334	9	0.00318
XP_010967350.1	ITIH4	104921.2	0.221	1	3	1.23	0.251	9	0.02605
XP_010971058.1	SIGLEC1	182903.2	0.008	1	1	1.42	0.329	9	0.002322
XP_010951346.1	SEC13	36476.59	0.229	5	11	1.23	0.193	9	0.006339
XP_010959957.1	EI24	39066.71	0.024	1	1	1.39	0.244	9	0.00061
XP_010956004.1	P3H3?	67698.96	0.037	2	2	1.29	0.317	9	0.02245
XP_010965673.1	CHMP5	24638.37	0.178	3	3	1.22	0.207	9	0.0109
XP_010948432.1	POLR2I	12407.73	0.107	1	2	1.22	0.262	9	0.03294
XP_010967078.1	WARS1	53758.9	0.321	14	29	1.26	0.207	9	0.004415
XP_010950686.1	SELENOH	13409.22	0.287	3	5	1.21	0.239	9	0.02572
XP_010949752.1	ALG11	56351	0.022	1	1	1.56	0.563	9	0.008799
XP_010967348.1	ITIH3	100027.9	0.122	10	13	1.28	0.334	9	0.04066
XP_010957743.1	MRFAP1	14809.44	0.063	1	1	1.22	0.269	9	0.04673
XP_010955926.1	CEP104	105034.8	0.008	1	1	1.76	0.144	9	3.58E-08
XP_010949761.1	MRPS31	43875.61	0.021	1	1	1.3	0.221	9	0.001946
XP_010950039.1	DHCR24	60864.28	0.041	2	2	1.25	0.296	9	0.03992
XP_010947651.1	MRPS36	11395.85	0.255	3	3	1.32	0.36	9	0.02504
XP_010958620.1	PLA2G6	84962.69	0.016	1	1	2.49	2.008	9	0.03264
XP_010963951.1	VTA1	33866.89	0.056	2	2	1.36	0.3	9	0.003631
XP_010963542.1	HYPK	13624.85	0.14	1	1	1.41	0.367	9	0.008827
XP_010962227.1	STAG2	142350.9	0.032	1	1	1.26	0.17	9	0.0009026

XP_010958787.1	MED1	62304.74	0.014	1	1	1.41	0.313	9	0.003973
XP_010951822.1	REXO2	23950.15	0.133	4	5	1.21	0.21	9	0.01423
XP_010949954.1	TPD52	19998.32	0.201	4	8	1.24	0.242	9	0.01438
XP_010959231.1	PSMF1	29959.91	0.166	4	6	1.21	0.211	9	0.01604
