# Supplementary material for: Screening and Identification of Differential Ovarian Proteins before and after Induced Ovulation via Seminal Plasma in Bactrian Camels
Source: Animals (Basel). 2021 Dec 9;11(12):3512. doi: 10.3390/ani11123512 (PMC8698062; doi:10.3390/ani11123512)
Supplement: Supplementary file 1 [file animals-11-03512-s001.zip › Table S2.pdf]

Table S2 Bactrian camel SP-induced ovulation down-regulated differential protein list of ovarian tissues

Protein_ID	gene	Mass	Protein_Coverage	Uniq_Pep_Num	Uniq_Spec_Num	Mean_Ratio_treated-VS-control	SD_treated-VS-control	Quant_No_treated-VS-control	Pvalue_treated-VS-control
XP_010956945.1	ELMO2	82942.4	0.082	5	7	0.83	0.116	9	0.003508
XP_010947665.1	LAP2	146426	0.015	2	2	0.72	0.321	9	0.04316
XP_010960214.1	NAT14	21915.6	0.049	1	1	0.74	0.059	9	2.85E-06
XP_010956110.1	ESPN	57139.6	0.01	1	1	0.79	0.188	9	0.008237
XP_010964587.1	LPAAT1	31814.11	0.022	1	1	0.76	0.364	9	0.04943
XP_010968477.1	DT	413332.6	0.048	5	6	0.81	0.125	9	0.002864
XP_010949080.1	S7	27811.5	0.103	2	2	0.83	0.151	9	0.00947
XP_010949603.1	NEFL	100795	0.025	1	1	0.78	0.292	9	0.04042
XP_010946614.1	Wnt2b	39111.7	0.088	3	5	0.83	0.197	9	0.03151
XP_010951710.1	MCAM	71335.16	0.097	6	8	0.79	0.195	9	0.01312
XP_010944991.1	Rint1	91359.21	0.009	1	1	0.75	0.175	9	0.003451
XP_010945878.1	ARF5	20612.61	0.267	1	1	0.75	0.293	9	0.03062
XP_010951309.1	BRPF1	138985.9	0.006	1	1	0.81	0.243	9	0.03379
XP_010962783.1	WASH1	50649.3	0.062	3	3	0.68	0.237	9	0.004151
XP_010950191.1	c-Rel	66234.18	0.015	1	2	0.77	0.273	9	0.04639
AMT92207.1		14686.93	0.167	1	3	0.77	0.268	9	0.03278
XP_010962636.1	KRT1	58011.2	0.064	2	2	0.66	0.297	9	0.008686
XP_010946694.1	TSTD3	18132.4	0.05	1	1	0.83	0.13	9	0.005728
XP_010964105.1	CDK19	55443.13	0.033	2	2	0.83	0.144	9	0.009688
XP_010964436.1	PTPP	41532.84	0.038	1	1	0.74	0.333	9	0.04639

XP_010964602.1	FST	40088.26	0.064	2	2	0.75	0.276	9	0.02372
XP_010969598.1	EXOC6	93167.6	0.016	1	1	0.64	0.241	9	0.003076
XP_010945841.1	TMEM209	62909.49	0.048	2	2	0.81	0.108	9	0.00133
XP_010967364.1	LOC105080173	67384.86	0.017	1	1	0.81	0.224	9	0.0369
XP_010965490.1	ISPS	44892.95	0.028	1	1	0.83	0.153	9	0.01316
XP_010964972.1	Di-Ras1	22637.58	0.03	1	1	0.71	0.249	9	0.009938
XP_010953311.1	DMTN	43191.91	0.066	2	3	0.78	0.131	9	0.002007
XP_010966632.1	SLMAP	94038.99	0.023	2	4	0.78	0.179	9	0.008514
XP_010955588.1	MLC1	41224.34	0.021	1	1	0.79	0.155	9	0.004319
XP_010951582.1	SORBS2	159650.3	0.048	6	14	0.71	0.274	9	0.01022
XP_010969476.1	Sorbs1	119493.2	0.152	14	20	0.78	0.202	9	0.0113
XP_010967575.1	ATM	355532.7	0.011	3	3	0.83	0.144	9	0.009045
XP_010968337.1	SGCD	32390.93	0.173	5	11	0.79	0.117	9	0.001075
XP_010957415.1	LIMS1	45958.94	0.194	2	3	0.83	0.227	9	0.04161
XP_010950554.1	GK	60371.26	0.017	1	1	0.79	0.218	9	0.0199
XP_010949908.1	LRR63	79248.87	0.013	1	1	0.81	0.222	9	0.02956
XP_010961882.1	MVK	42967.28	0.109	3	4	0.79	0.04	9	6.35E-07
XP_010958610.1	CK1	47243.1	0.061	2	2	0.77	0.137	9	0.00197
XP_010944186.1	CCK	12824.42	0.078	1	1	0.67	0.407	9	0.01974
XP_010967324.1	ALCAM	65458.36	0.062	3	3	0.77	0.08	9	4.72E-05
XP_010949050.1	MANA	56890.72	0.022	1	1	0.74	0.274	9	0.02264
XP_010953662.1	TRIM47	93000.89	0.055	3	3	0.81	0.086	9	0.000267
XP_010971659.1	MED17	73130.66	0.017	1	1	0.78	0.1	9	0.000375
XP_010968301.1	GPX3	25687.08	0.319	6	20	0.71	0.165	9	0.002564
XP_010962933.1	TSPAN11	28692.44	0.028	1	1	0.71	0.173	9	0.00181
XP_010963840.1	L3MBTL3	86760.43	0.016	1	1	0.82	0.152	9	0.009266

XP_010966803.1	GGTB	61885.21	0.016	1	1	0.8	0.072	9	5.37E-05
XP_010946683.1	P400	302734	0.008	2	2	0.74	0.127	9	0.000675
XP_010945936.1	TESTIN	49796.58	0.154	5	6	0.81	0.129	9	0.002974
XP_010944878.1	PRELP	44539.68	0.497	20	160	0.83	0.208	9	0.03446
XP_010954554.1	LMOD1	55356.34	0.114	6	13	0.79	0.148	9	0.003457
XP_010950489.1	TINAGL1	53610.47	0.148	5	11	0.78	0.165	9	0.005322
XP_010963654.1	TNFAIP8L3	24997.11	0.037	1	1	0.77	0.153	9	0.003517
XP_010959025.1	HOXD8	12870.67	0.094	1	2	0.82	0.205	9	0.03127
XP_010955618.1	SCO2	29996.52	0.071	1	1	0.75	0.301	9	0.04767
XP_010959215.1	ZCCHC3	30211.72	0.037	1	1	0.78	0.241	9	0.02549
XP_010949354.1	septin-4	38879.85	0.073	1	1	0.78	0.095	9	0.00027
XP_010970276.1	NIPSNAP1	18082.21	0.248	1	1	0.81	0.088	9	0.000431
XP_010960883.1	NUMB	65090.51	0.042	2	2	0.76	0.144	9	0.002869
XP_010963168.1	ZBED5	80064.88	0.009	1	1	0.69	0.534	9	0.03582
XP_010957046.1	DHX35	79486.28	0.017	1	1	0.64	0.147	9	0.000416
XP_010949660.1	PATZ	72620.88	0.018	1	1	0.81	0.187	9	0.01621
XP_010955047.1	ALDH3B1	52474.8	0.026	1	1	0.71	0.143	9	0.000533
XP_010963746.1	LAMA4	205018.3	0.104	18	23	0.79	0.136	9	0.002195
XP_010955670.1	RPL11	19194.19	0.216	3	8	0.8	0.175	9	0.01065
XP_010954506.1	NR5A1	47909.26	0.026	1	1	0.5	0.222	9	0.001838
XP_010962989.1	PDX1	54447.78	0.044	2	2	0.83	0.232	9	0.03642
XP_010958013.1	CLYBL	38249.28	0.037	1	1	0.79	0.12	9	0.001114
XP_010959401.1	MYH11	226805.5	0.346	59	190	0.72	0.147	9	0.000825
XP_010963102.1	ABCC8	178015.3	0.003	1	1	0.74	0.254	9	0.02385
XP_010956520.1	VPS4A	95150.34	0.01	1	1	0.56	0.295	9	0.00617
XP_010947851.1	GLRX1	12125.23	0.113	2	3	0.83	0.147	9	0.008771

XP_010952841.1	LIMS2	40680.32	0.158	1	1	0.79	0.247	9	0.03462
XP_010962644.1	KRT1	62465.73	0.088	3	9	0.71	0.19	9	0.004437
XP_010959856.1	PPP1R10	98159.5	0.011	1	1	0.59	0.319	9	0.009315
XP_010952947.1	C2orf76	14880.78	0.056	1	1	0.77	0.261	9	0.03667
XP_010944727.1	Ybx3	53131.57	0.061	1	1	0.69	0.3	9	0.029
XP_010971132.1	MVI	146165.7	0.126	14	20	0.81	0.095	9	0.000411
XP_010967959.1	ANAPC7	67163.91	0.015	1	1	0.71	0.111	9	0.000111
XP_010967496.1	LAMB2	202622.7	0.172	27	38	0.82	0.125	9	0.002692
XP_010967757.1	MYZAP	53943.65	0.03	1	1	0.8	0.214	9	0.02093
ADG03660.1		11895.45	0.154	1	3	0.72	0.355	9	0.03764
XP_010960955.1	ZNF410	52594.04	0.031	1	1	0.83	0.241	9	0.04347
XP_010947392.1	PFDN6	14555.79	0.202	3	8	0.8	0.147	9	0.005876
XP_010949648.1	smoothelin	97707.59	0.102	7	7	0.7	0.246	9	0.0063
XP_010972181.1	PYCR2	27296.21	0.281	2	3	0.65	0.218	9	0.001938
XP_010947544.1	MSH3	128147.3	0.021	2	2	0.82	0.067	9	6.54E-05
XP_010958915.1	PNPLA7	150701.1	0.022	1	1	0.76	0.172	9	0.006996
XP_010961454.1	CAMK2G	64653.61	0.113	2	3	0.75	0.138	9	0.000923
XP_010966335.1	KRT17	40049.28	0.104	1	1	0.76	0.141	9	0.001401
XP_010967644.1	PPBP	13080.11	0.067	1	2	0.79	0.301	9	0.0447
XP_010954259.1	DDX31	80431.49	0.018	1	1	0.79	0.237	9	0.02116
XP_010966285.1	MRPL19	33282.16	0.038	1	1	0.79	0.063	9	1.85E-05
XP_010949518.1	ZER1	85836.48	0.018	1	1	0.83	0.18	9	0.02163
XP_010952232.1	MSTO1	63046.86	0.012	1	1	0.76	0.122	9	0.00093
XP_010952399.1	KCR1	56269.34	0.038	2	2	0.78	0.178	9	0.00529
XP_010961773.1	RPS27L	9794.985	0.155	1	3	0.78	0.206	9	0.01091
XP_010963398.1	RyR3	557798.3	0.002	1	1	0.66	0.328	9	0.01306

XP_010949936.1	XPO4	127611.2	0.006	1	1	0.76	0.172	9	0.003546
XP_010947126.1	Desmin	51694.15	0.522	22	107	0.77	0.205	9	0.01074
XP_010968782.1	FBXO7	62252.79	0.073	3	4	0.79	0.064	9	2.82E-05
XP_010960563.1	HSPA2	70059.92	0.341	13	23	0.82	0.105	9	0.001554
XP_010970457.1	PGPEP1	24549.04	0.037	1	1	0.7	0.23	9	0.009259
XP_010953031.1	MYO7	223955.3	0.008	1	1	0.8	0.252	9	0.04165
XP_010953249.1	Lamc1	182806.8	0.185	23	54	0.8	0.184	9	0.01568
XP_010945811.1	A4D1P6	84239.16	0.048	4	4	0.82	0.111	9	0.001512
XP_010959383.1	NOB1	55164.32	0.018	1	1	0.61	0.242	9	0.003384
XP_010948772.1	ZNF326	63045.64	0.045	2	2	0.83	0.13	9	0.004814
XP_010969708.1	TTC21B	142356.5	0.007	1	1	0.83	0.12	9	0.003472
XP_010951841.1	CBL	96686.06	0.013	1	1	0.83	0.102	9	0.001525
XP_010956347.1	RMC1	68763.64	0.02	1	1	0.74	0.138	9	0.000765
XP_010952456.1	OR	36908.18	0.022	1	1	0.69	0.497	9	0.03684
XP_010955494.1	TRIM32	73409.94	0.017	1	1	0.63	0.212	9	0.001277
XP_010972093.1	LIN9	52456.01	0.011	1	1	0.59	0.323	9	0.009139
XP_010948554.1	SHPK	56500.32	0.041	1	1	0.78	0.061	9	1.25E-05
XP_010968229.1	EPC1	80796	0.012	1	1	0.79	0.189	9	0.009126
XP_010959749.1	PBX2	40567.93	0.041	1	1	0.83	0.123	9	0.003981
XP_010968409.1	SVEP1	419995.7	0.003	1	1	0.73	0.328	9	0.0254
XP_010960112.1	DCTN4	53235.98	0.141	5	8	0.83	0.1	9	0.001168
XP_010967344.1	PRKCD	78578.35	0.044	2	2	0.8	0.118	9	0.001884
XP_010963941.1	HEBP2	22287.8	0.212	4	9	0.83	0.142	9	0.006272
XP_010950231.1	EML6	218751.5	0.007	1	1	0.28	0.208	9	0.000207
XP_010953748.1	DDT	13506.16	0.371	4	24	0.76	0.138	9	0.00138
XP_010960174.1	LENG8	90972.65	0.011	1	1	0.77	0.248	9	0.03507

XP_010956791.1	RPRD2	100706.2	0.019	2	2	0.72	0.046	9	2.52E-07
XP_010960017.1	ECHDC3	33467.27	0.029	1	3	0.77	0.192	9	0.009866
XP_010965034.1	PLIN4	141622.1	0.034	4	5	0.8	0.181	9	0.0122
XP_010972498.1	H2A	14036.88	0.515	1	3	0.71	0.305	9	0.01351
XP_010972224.1	BAG2	23756.22	0.123	3	3	0.72	0.077	9	1.65E-05
XP_010962375.1	FLNA	283276	0.45	88	341	0.82	0.206	9	0.02941
XP_010954253.1	HMCN2	421525	0.006	2	2	0.74	0.153	9	0.001832
XP_010966017.1	PLEKHH2	170067.8	0.003	1	1	0.74	0.114	9	0.000321
XP_010951950.1	OSTC	16899.85	0.081	1	1	0.8	0.221	9	0.02901
XP_010963267.1	CAVIN3	27350.33	0.41	9	15	0.77	0.23	9	0.01442
XP_010970704.1	AHSP	10757.44	0.141	1	1	0.8	0.174	9	0.007832
XP_010970421.1	GTPBP3	52882.34	0.028	1	1	0.83	0.139	9	0.01039
XP_010961153.1	ARID4B	147776.9	0.013	1	1	0.67	0.157	9	0.000873
XP_010944770.1	ART4	36899.12	0.034	1	2	0.81	0.159	9	0.01023
XP_010953081.1	TINF2	47835.49	0.014	1	1	0.77	0.185	9	0.008913
XP_010966482.1	CTU2	56021.09	0.022	1	1	0.66	0.136	9	0.000291
XP_010955400.1	MEN1	66197.6	0.02	1	1	0.81	0.198	9	0.01713
XP_010967476.1	RNF123	150103.7	0.013	2	2	0.76	0.176	9	0.008168
XP_010972376.1	MARK1	73861.14	0.014	1	2	0.64	0.187	9	0.001625
XP_010968641.1	CCDC127	27522.46	0.03	1	1	0.73	0.261	9	0.01272
