# Supplementary figures and images for: Screening and Identification of Differential Ovarian Proteins before and after Induced Ovulation via Seminal Plasma in Bactrian Camels
Source: Animals (Basel). 2021 Dec 9;11(12):3512. doi: 10.3390/ani11123512 (PMC8698062; doi:10.3390/ani11123512)

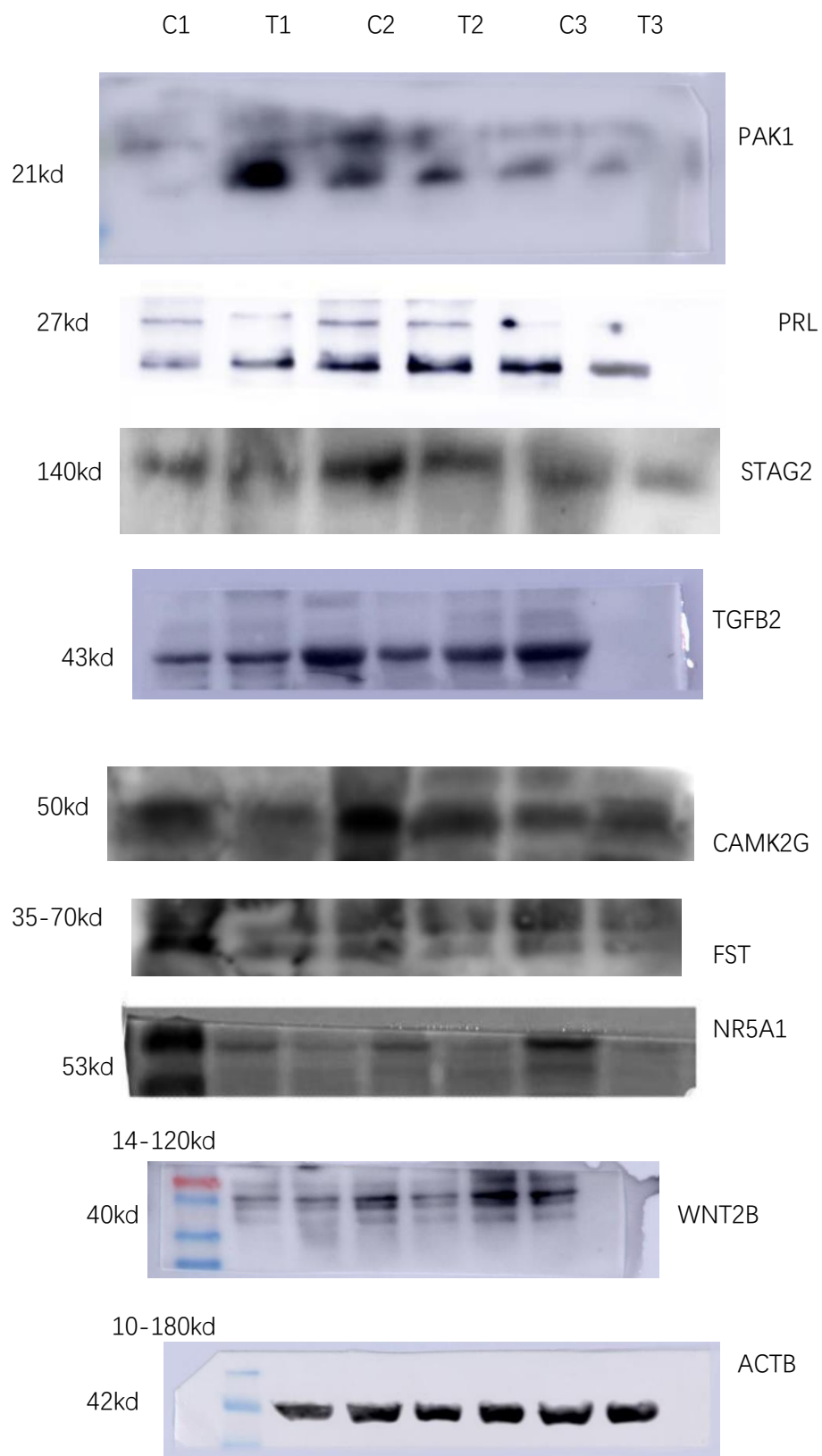

Supplement: Supplementary file 1 [file animals-11-03512-s001.zip › Figure S2.pdf]

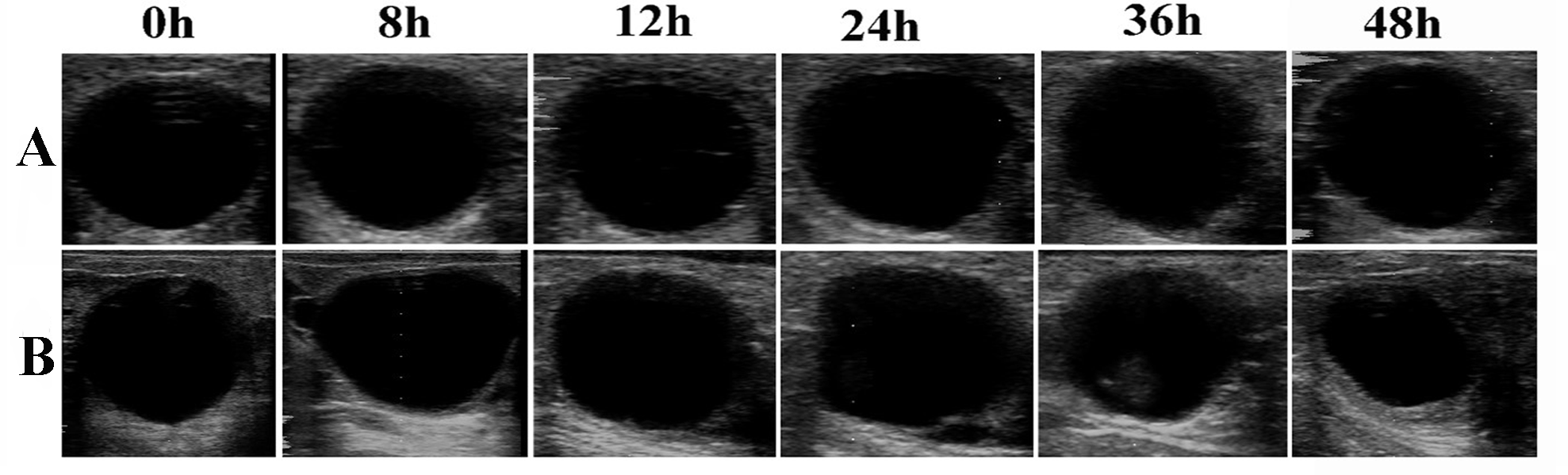

Supplement: Supplementary file 1 [file animals-11-03512-s001.zip › FigureS1.tif]
